# Extracellular vesicles from chemotherapy-induced senescent tumor cells reprogram hepatic phospholipid metabolism to promote pancreatic cancer liver metastasis

**DOI:** 10.1186/s13046-026-03741-3

**Published:** 2026-05-22

**Authors:** Bai’an Tao, Yecheng Xu, Wenteng Miao, Zhenmei Chen, Jichun Gu, Yujie Guo, Linjie Chen, Yang Di, Hang He, Chen Jin, Ji Li, Deliang Fu

**Affiliations:** 1 Department of Pancreatic Surgery, Huashan Hospital, Fudan University, No. 12, Middle Urumqi Road, Jing’an District, Shanghai, 200040 China; 2 Department of General Surgery, Huadong Hospital, Fudan University, Shanghai, 20040 China

**Keywords:** Pancreatic cancer liver metastasis, Neoadjuvant chemotherapy, Senescence, Extracellular vesicles, Hepatic phospholipid metabolism

## Abstract

**Background:**

Pancreatic ductal adenocarcinoma (PDAC) is a highly metastatic disease, with over one-third of patients developing liver metastasis following neoadjuvant chemotherapy (NAC). This study aimed to investigate how the effect of chemotherapy on the primary tumor shapes the hepatic pre-metastatic niches (PMNs), which remains poorly understood.

**Methods:**

RNA sequencing was performed in a PDAC cohort to identify transcriptomic features associated with metastasis. We employed orthotopic and liver metastatic tumor models in mice to investigate the effects of extracellular vesicles derived from chemotherapy-treated PDAC cells, referred to as senescence-associated extracellular vesicles (S-EVs), on the hepatic PMNs. Mechanistically, we integrated proteomics and metabolomics to elucidate the molecular mechanisms by which S-EVs mediate hepatic metabolic reprogramming. Further, we utilized hepatocyte-specific Ache-knockout mice to validate the molecular mechanisms through which S-EVs promote liver metastasis in vivo.

**Results:**

Chemotherapy-induced senescence in primary tumors correlated with PDAC liver metastasis and poor prognosis. S-EVs facilitated PMNs formation by promoting hepatic phosphatidylcholine (PC) accumulation. Mechanistically, S-EV-delivered hnRNPA1 stabilized AChE mRNA, thereby driving PC accumulation in the liver. In vitro co-culture system, PC depletion in hepatocytes successfully restored the impaired cytotoxicity of CD8⁺ T cells caused by S-EVs. Consistently, hepatocyte-specific Ache ablation in vivo restored S-EV-impaired CD8⁺ T cells cytotoxicity. Inhibition of AChE with pyridostigmine remodeled the hepatic PMNs and suppressed liver metastasis.

**Conclusions:**

S-EVs from chemotherapy-induced senescent PDAC cells reshaped hepatic PMNs through AChE-dependent PC accumulation, thereby impairing CD8⁺ T cell cytotoxicity and promoting PDAC liver metastasis. Targeting AChE with pyridostigmine represented a promising strategy to prevent metastasis and augment the therapeutic efficacy of NAC.

**Supplementary Information:**

The online version contains supplementary material available at 10.1186/s13046-026-03741-3.

## Background

Pancreatic ductal adenocarcinoma (PDAC) is a highly lethal malignancy [1, 2], and approximately 80% of patients are diagnosed at an advanced, surgically unresectable stage, necessitating neoadjuvant chemotherapy (NAC) [3–5]. Although NAC improves the survival outcomes of patients with PDAC, the 5-year survival remains below 15%, and liver metastasis is the leading cause of mortality [6–9]. More than one-third of PDAC patients receiving NAC develop postoperative liver metastasis [10, 11]. Therefore, there is an urgent need to identify therapeutic targets and develop strategies to potentiate neoadjuvant chemotherapy in PDAC and inhibit liver metastasis.

Mounting evidence indicates that chemotherapy remodels distant organs and promote the formation of pre-metastatic niches (PMNs) [12, 13]. NAC-activated endothelial EVs induce CCL2-mediated recruitment of Ly6C⁺CCR2⁺ monocytes to remodel the pulmonary PMNs in breast cancer [14]. Additionally, chemotherapies of cisplatin and paclitaxel have been shown to upregulate VEGFR1 on lung endothelial cells, facilitating the seeding of circulating tumor cells [15]. In PDAC, primary tumors can recruit CD11b⁺ dendritic cells and establish an immunosuppressive milieu via PD-L2–mediated CD8⁺ T-cell suppression and Treg expansion, thus promoting liver metastasis [16]. However, the impact of NAC on remodeling the hepatic PMNs in PDAC remains unknown.

Chemotherapy has been shown to induce tumor cell senescence characterized by stable growth arrest^9–12^ [17, 18]. Senescent tumor cells remain metabolically active and influence distant organs through the release of extracellular vesicles (EVs). EVs released from chemotherapy-induced senescent nasopharyngeal carcinoma cells have been reported to carry circWDR37, thereby promoting distant metastasis [19]. Additionally, chemotherapy-induced senescent colorectal cancer cells have been shown to release increased levels of EVs, which in turn promote tumor progression and metastasis through SERPINE1-mediated NF-κB p65 nuclear translocation [20]. We previously showed that PDAC-derived EVs are important mediators of hepatic PMNs formation, but the specific role of EVs from NAC-induced senescent PDAC cells in remodeling the hepatic PMNs remains unclear [21]. Moreover, several studies have reported that tumor-derived EVs modulate hepatic immune responses by reprogramming liver metabolism [22–25]. In colorectal cancer, tumor-derived EVs enriched in prosteatogenic lipids are taken up by Kupffer cells, promoting tumor necrosis factor-α (TNF-α) secretion and facilitating liver metastasis [26]. In addition, PDAC-derived EVs are important regulators of hepatic lipid metabolic rewiring, thereby fostering a pro-inflammatory liver microenvironment [27]. On this basis, we hypothesized that EVs released from NAC-induced senescent PDAC cells promote the formation of hepatic pre-metastatic niches by reprogramming liver metabolism.

In this study, we found that NAC-induced senescence of primary lesions was associated with liver metastasis and poor prognosis in PDAC patients. S-EVs released from senescent PDAC cells promoted liver metastasis through reprogramming the metabolism in the liver, thereby impairing CD8⁺ T cell anti-tumor immunity. Mechanistically, hnRNPA1, delivered by S-EVs, increased AChE expression and causing PC accumulation. Inhibition of AChE with pyridostigmine restored CD8⁺ T cell anti-tumor immunity, and suppressed liver metastasis in vivo, appearing as a therapeutic option to improve NAC efficacy.

## Methods

### Patients

The study involved a well-monitored cohort of 94 PDAC patients from Huashan Hospital, Fudan University. All samples were obtained from patients who underwent surgical resection after neoadjuvant chemotherapy with gemcitabine plus nab-paclitaxel between 1 January 2018 and 31 December 2022. All cases were pathologically confirmed as pancreatic ductal adenocarcinoma by two experienced pathologists. All patients underwent follow-up, including serial imaging assessments. Based on the presence or absence of liver metastasis within 6 months after surgery, patients were classified into the liver metastasis (LM) group (*n* = 25) and the non-liver metastasis (nLM) group (*n* = 69). Furthermore, we additionally included 65 patients who underwent primary pancreatic cancer resection following neoadjuvant chemotherapy in our department, subsequently developed liver metastases, and underwent surgical resection of these hepatic lesions. All specimens were preserved in formalin or stored at –80 °C for downstream analyses. Written informed consent was obtained from all patients for their data and samples to be used for research purposes. The study was approved by the Ethics Committee of Huashan Hospital, Fudan University.

### Animal experiments

Animal experiments were designed and performed according to ARRIVE reporting guidelines. C57BL/6 J, Albumin-cre and Ache-flox mice (6 weeks of age) were obtained from GemPharmatech Laboratory (Nanjing, China). All mice were housed in ventilated caging units in the Shanghai Cancer Center Specific Pathogen–Free facility with standard housing and husbandry and free access to food and water. To investigate how chemotherapy affects the primary tumor and subsequent liver metastasis, orthotopic pancreatic tumors were established by surgically exposing the pancreatic tail and injecting 2 × 10^5^ KPC cells in 20 µL Matrigel (1:1) into the pancreatic parenchyma of C57BL/6 J mice under isoflurane anesthesia. Seven days after orthotopic cell implantation, mice were randomized to receive a senescence-inducing low-dose chemotherapy regimen. Mice were administered Gemcitabine (25 mg/kg) and Nab-paclitaxel (10 mg/kg) via intraperitoneal injection every 3 days (q3d). This low-dose regimen was designed to induce tumor cell senescence while minimizing massive cell death. On day 14, mice were anesthetized, and the orthotopic primary tumors were surgically resected to simulate the clinical scenario of pancreaticoduodenectomy following NAC. Liver metastasis was then induced by splenic subcapsular injection of KPC cells to generate a PDAC liver-metastasis model. To examine the formation of a pro-metastatic hepatic microenvironment, EVs from orthotopic pancreatic tumors and KPC cells treated with AG were administered via the tail vein for 2 weeks before the induction of liver metastasis. CD8⁺ T cell depletion was induced by anti-CD8α (200 µg i.p. every 3 days, Monoclonal Antibody 2.43, Functional Grade, In Vivo PLATINUM™—C2837-1MG). Matched mouse IgG2b isotype was given on the same schedule. Ache^flox/flox^ (Ache^f/f^) mice with C57BL/6 genetic background were generated by targeting the predicted promoter region and exon 2–3 of the Ache-201 (ENSMUST00000024099.10) transcript (encoding the Ache) using a Cre-LoxP system. Ache^f/f^ male mice were crossed with albumin-Cre transgenic female mice to produce hepatocyte-specific Ache knockout (Ache^△hepa^) mice. To assess the therapeutic efficacy of pyridostigmine in preventing liver metastasis, we established a murine liver metastasis model. Mice were first pre-educated with tail vein injections of S-EVs or N-EVs (10 µg every 2 days for 2 weeks) to induce pre-metastatic niche formation. Subsequently, tumor cells were injected intrasplenically to establish metastases. Starting from day 14, mice were randomized to receive intragastric administration of either pyridostigmine (15 mg/kg) or an equivalent volume of vehicle (PBS) every 3 days. Six-week-old male mice were used for all experiments. KPC cells were used to establish both orthotopic PDAC tumors and liver metastasis models. Mice were humanely killed by CO₂ inhalation before tumor removal. Orthotopic PDAC tumors and liver metastases were harvested for subsequent analyses. All animals were housed and maintained in the Laboratory Animal Center of Fudan University. All animal study procedures were approved by the Ethics Committee of Huashan Hospital, Fudan University.

### Cell lines and culture

KPC mice-derived PDAC cell line FC1199, referred to as KPC cells, were derived from primary KPC tumors obtained from Kras^G12D/+^; TP53^R172H/+^; Pdx-1-Cre^+/+^ mice. Panc02 cells were purchased from the National Infrastructure of Cell Line Resource. Primary mouse hepatocytes (PMHs) were isolated from 6-week-old C57BL/6 mice by liver perfusion with Ca^2^⁺/Mg^2^⁺-free HBSS followed by type IV collagenase digestion, mechanical dissociation, filtration, and low-speed centrifugation. Viable hepatocytes were further purified by Percoll gradient centrifugation. PDAC cell lines and PMHs were cultured in DMEM with 10% FBS.

CD8^+^ T cells were negatively selected with a CD8^+^ T cell isolation kit (Miltenyi). Mouse spleens were harvested into ice-cold PBS. Single-cell suspensions were prepared by passing tissue through a 70-µm strainer and centrifuging (400 g, 5 min, 4 °C). RBCs were lysed with ACK (min) and quenched with PBS. Cells were resuspended in autoMACS Running Buffer (Miltenyi, 130–091–221), adjusted to 1 × 10^7^ cells/100 µL, incubated with CD8a (Ly-2) MicroBeads (Miltenyi, 130–117-044; 10 µL/1 × 10^7^ cells) for 15 min at 4 °C, washed, and selected on an autoMACS separator. CD8⁺ T cells were seeded into 24-well plates pre-coated with anti-CD3 and anti-CD28 antibodies and cultured in RPMI-1640 supplemented with 10% FBS and IL-2. All cells were cultured at 37 °C in a humidified atmosphere containing 5% CO_2_.

### Extracellular vesicles purification and characterization

Sequential centrifugation was used to purify all the EVs in our experiments. To extract EVs from orthotopic pancreatic tumors, freshly excised tumors were rinsed in ice-cold PBS, minced into ~ 1–2 mm^3^ fragments and incubated in serum-free, exosome-depleted medium for 24 h; conditioned media were sequentially centrifuged at 300 × g for 10 min, 2,000 × g for 20 min and 10,000 × g for 30 min, passed through a 0.22-µm filter, and EVs were pelleted by ultracentrifugation at 100,000 × g for 70 min at 4 °C, washed once in PBS (100,000 × g, 70 min) and resuspended in PBS for downstream analyses. For the EVs from KPC cells, culture medium underwent a two-step centrifugation to first remove live cells, potential apoptotic bodies, and large cell debris.: 500 × g for 10 min, and 12,000 × g for 20 min. The EV-containing pellets were then gathered by centrifuging at 100,000 × g for 70 min. Following a PBS wash, the pellets underwent ultracentrifugation using a Beckman 70Ti rotor. To generate senescence-associated extracellular vesicles (S-EVs), PDAC cells were first induced to senesce by treatment with low-dose Gemcitabine (50 nM) and Paclitaxel (10 nM) for 48 h. Subsequently, the drug-containing medium was removed, cells were washed, and fresh exosome-free medium was added. S-EVs were then isolated from the conditioned medium collected after an additional 48–72 h of culture. The quality, size, and particle number of EVs were characterized by transmission electron microscope (TEM) and Nano-particle Tracking Analysis (NTA). The protein concentration for each EVs sample was determined by BCA (Pierce, Thermo Fisher Scientific). All the purified EVs pellets were resuspended in PBS and stored at –80 °C.

### Statistical analysis

Statistical analyses were performed using GraphPad Prism software (GraphPad Software, San Diego, CA). Differences between groups were assessed using Student’s t-test, ANOVA, or the Chi-square (χ^2^) test, as appropriate. Correlations between continuous variables were analyzed using linear regression and Pearson’s correlation coefficient. Data are presented as mean ± SD or mean ± SEM. Statistical significance is indicated as follows: * P < 0.05, ** *P* < 0.01, *** *P* < 0.001, and **** *P* < 0.0001.

Additional details regarding materials and methods are provided in the Supplementary Materials.

## Results

### NAC-induced senescence of primary PDAC was associated with liver metastasis and poor prognosis

**Fig. 1:**
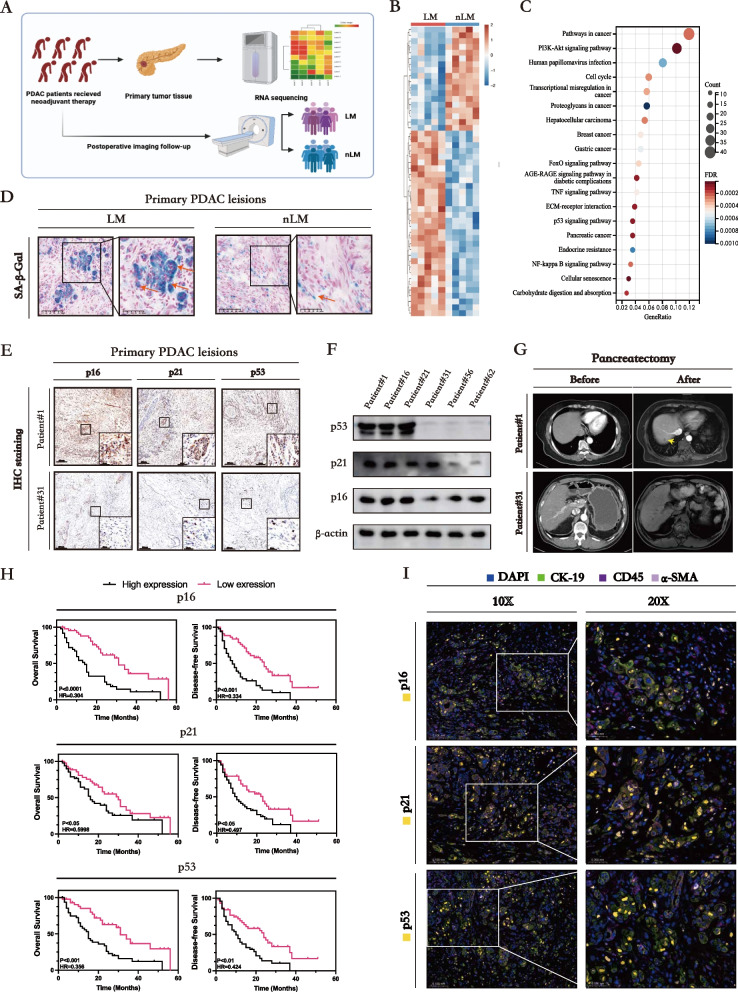
NAC-induced senescence of primary PDAC was associated with liver metastasis and poor prognosis. **A** Schematic workflow of the study design. Primary tumor tissues were collected from PDAC patients treated with neoadjuvant chemotherapy (NAC) followed by RNA sequencing and clinical follow-up. Patients were stratified into liver metastasis (LM, *n* = 25) and non-liver metastasis (nLM, *n* = 69) groups based on liver metastasis within 6 months post-surgery. **B** Heatmap showing differentially expressed genes (DEGs) between the LM and nLM groups. **C** KEGG pathway enrichment analysis of the identified DEGs, highlighting the enrichment of senescence-related pathways. **D** Representative images of senescence-associated β-galactosidase (SA-β-gal) staining in primary PDAC tissues from the LM and nLM groups. Senescent cells are stained in dark blue. **E** Representative immunohistochemical (IHC) staining of senescence markers (p16, p21, and p53) in resected PDAC tissues after NAC treatment. **F** Immunoblot analysis of p16, p21, and p53 expression levels in representative PDAC tissue samples. **G** Representative contrast-enhanced MRI scans showing hepatic metastatic lesions (yellow arrows) in post-NAC PDAC patients. **H** Kaplan–Meier curves for disease-free survival (DFS) and overall survival (OS) of NAC-treated PDAC patients, stratified by the median expression levels of p16, p21, and p53. **I** Representative multiplex immunohistochemistry (mIHC) images showing the cellular localization of senescence markers. Tumor cells are marked by CK19 (green), immune cells by CD45 (purple), fibroblasts by α-SMA (pink), and senescence markers (p16/p21/p53) are shown in yellow. Nuclei are stained with DAPI (blue)

Firstly, we collected tumor specimens from PDAC patients treated with NAC (Supplementary Fig. 1A). Baseline clinical characteristics are summarized in Table 1. Patients were categorized into the liver metastasis (LM) group and the non-liver metastasis (nLM) group based on the occurrence of liver metastasis within 6 months postoperatively [28, 29] (Fig. 1A). Next, we performed RNA sequencing on primary tumor samples and identified significant differences in transcriptomic profiles between the LM and nLM groups (Fig. 1B). The KEGG pathway enrichment analysis highlighted significant gene enrichment related to cellular senescence, p53, cell cycle, PI3K–AKT, AGE–RAGE and NF-κB pathways (Fig. 1C). TUNEL assay revealed no significant difference in the proportions of apoptotic cells between LM and nLM groups (Supplementary Fig. 1B). For further confirmation, SA-β-gal staining revealed that the proportion of positive cells in primary tumors was significantly higher in the LM group than in the nLM group (Fig. 1D). In addition, we evaluated the expression of senescence-associated markers (p16, p21, and p53) in primary tumors from NAC-treated PDAC patients (*n* = 94) and assessed liver metastasis status by contrast-enhanced MRI (Fig. 1E-G). Patients were stratified by the median expression of senescence markers; notably, the high-expression group exhibited significantly reduced overall survival (OS) and disease-free survival (DFS) compared to the low-expression group (Fig. 1H). Further multiplex IHC confirmed that senescence markers were mainly expressed in CK19⁺ PDAC tumor cells (Fig. 1I). To determine whether senescence was specifically induced by NAC, we analyzed the extent of senescence in the primary tumors of PDAC patients who underwent upfront resection (UR) compared to those in the NAC group. We found that the level of senescence in the primary tumors was significantly lower in the UR group than in the NAC group (Supplementary Fig. 1C). This indicates that senescence is rarely observed in the primary tumors of chemotherapy-naïve patients. Collectively, these findings identified the NAC-induced senescence of tumor cell as a defining feature of primary PDAC lesions associated with early liver metastasis, and poor survival.

**Table 1:** Baseline clinicopathological characteristics of pancreatic ductal adenocarcinoma patients receiving neoadjuvant chemotherapy

**Characteristics**		**nLM (*n* = 69)**	**LM (*n* = 25)**	***p*.value**
Age (years)		60.83	61.48	0.762
pre CA19-9 (U/mL)		240.90	222.75	0.867
pre CEA (U/mL)		6.63	4.81	0.621
Gender (%)	Female	28 (40.6)	12 (48.0)	0.52
	Male	41 (59.4)	13 (52.0)	
Artery invasion (%)	No	53 (76.8)	18 (72.0)	0.632
	Yes	16 (23.2)	7 (28.0)	
T status (%)	T1	15 (21.7)	0 (0.0)	0.08
	T2	25 (36.2)	13 (52.0)	
	T3	13 (18.8)	5 (20.0)	
	T4	16 (23.2)	7 (28.0)	
N status (%)	N0	37 (53.6)	8 (32.0)	0.116
	N1	21 (30.4)	9 (36.0)	
	N2	11 (15.9)	8 (32.0)	
M status (%)	M0	59 (85.5)	22 (88.0)	0.757
	M1	10 (14.5)	3 (12.0)	
Situation (%)	Head	42 (60.9)	16 (64.0)	0.93
	Tail	27 (39.1)	9 (36.0)	

*nLM* no liver metastasis, *LM* Liver metastasis, *pre CA19-9* preoperative CA19-9, *pre CEA* preoperative CEA

### Extracellular vesicles from senescent PDAC promoted liver metastasis

**Fig. 2:**
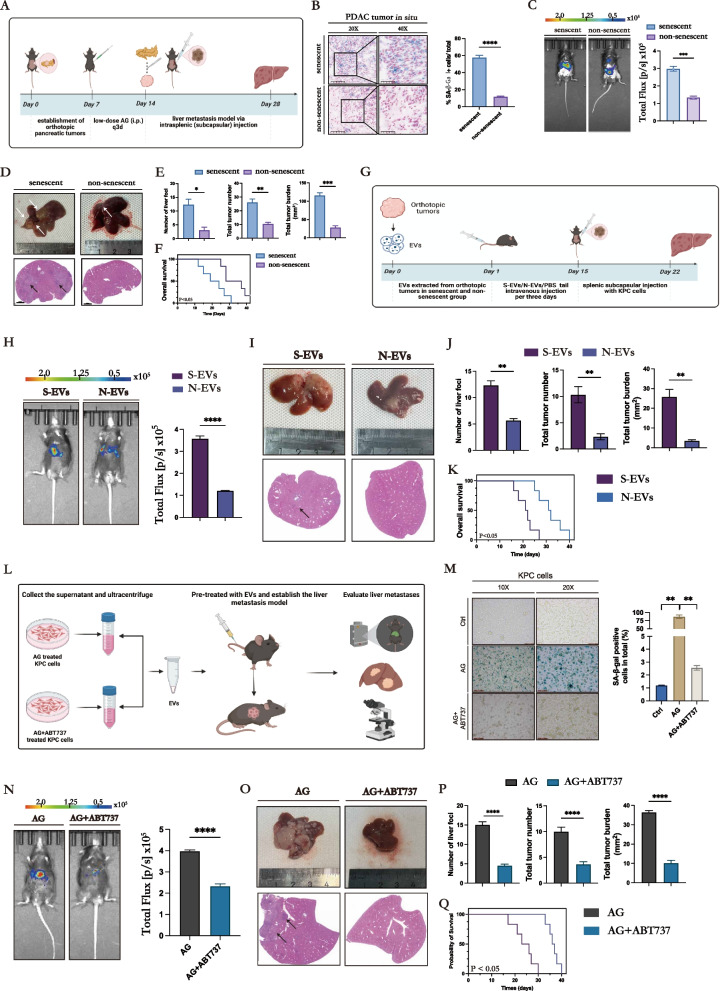
Extracellular vesicles from senescent PDAC promoted liver metastasis. **A** Schematic workflow for establishing the post-chemotherapy liver metastasis mouse model (*n* = 6 per group). **B** Representative images of SA-β-gal staining in resected orthotopic PDAC tumors (left) and quantitative analysis of SA-β-gal positive areas (right). Mice were stratified into senescent and non-senescent groups based on SA-β-gal activity. **C** Longitudinal monitoring of liver metastasis. Left: Representative in vivo bioluminescence images of mice from the senescent and non-senescent groups. Right: Quantification of the total flux from the liver region. Data are presented as mean ± SEM. **D**, **E** Representative macroscopic observations and H&E staining of liver metastases (**D**), followed by quantification of the metastatic burden (**E**). **F** Kaplan–Meier survival curves of mice in the senescent versus non-senescent groups (*P* < 0.5, log-rank test). **G** Schematic illustration of the experimental design: EVs were isolated from senescent (S-EVs) or non-senescent (N-EVs) orthotopic tumors and administered to naïve mice prior to metastasis induction (*n* = 6 per group). **H** Representative bioluminescence imaging (left) and signal quantification (right) of liver metastases in mice following preconditioning with S-EVs or N-EVs. **I**, **J** Assessment of liver metastatic burden. **I** Representative macroscopic images and H&E staining of liver tissues. **J** Statistical quantification of the metastatic nodules/tumor area. **K** Kaplan–Meier survival analysis of mice pretreated with S-EVs or N-EVs (*P* < 0.05, log-rank test). **L** Schematic workflow for examining the effect of senolysis on EV function. KPC cells were treated with AG (to induce senescence) combined with or without the senolytic agent ABT-737. **M** Representative images of SA-β-gal staining in orthotopic pancreatic tumor tissues from the control, AG, and AG + ABT-737 groups. **N**, **O** Representative in vivo bioluminescence images of liver metastasis in mice treated with EVs derived from the indicated KPC cell groups (AG vs. AG + ABT-737) (left), and quantification of the bioluminescence signals (right). **P** Representative macroscopic images and H&E staining of liver tissues from the indicated groups (left), and quantitative analysis of the metastatic burden (right). **Q** Kaplan–Meier overall survival curves of tumor-bearing mice treated with the indicated EVs (*P* < 0.05, log-rank test)

To further assess the role of NAC-induced senescence in liver metastasis in vivo, we first established orthotopic pancreatic tumors and treated mice with low-dose gemcitabine plus nab-paclitaxel (AG). The orthotopic tumors were then resected, and a liver metastasis model was generated as described (Fig. 2A). Based on SA-β-gal activity in the resected orthotopic tumor tissue, mice were stratified into senescent and non-senescent groups (n = 6 per group) (Fig. 2B). Mice with high SA-β-gal activity exhibited faster growth of hepatic metastases, with significantly greater metastatic burden and nodule number than those with low SA-β-gal activity (Fig. 2C–E). Moreover, overall survival was significantly shorter in the senescent group than in the non-senescent group (Fig. 2F). Given that EVs facilitate distant intercellular communication and metastatic progression via protein transport, we postulated that EVs derived from NAC-induced senescent PDAC cells contribute to the development of liver metastasis. We isolated EVs from orthotopic pancreatic tumors in the senescent and non-senescent groups and assessed their morphology and size distribution using transmission electron microscopy (TEM) and nanoparticle tracking analysis (NTA), respectively (Supplementary Fig. 2A–B). Next, both subsets of EVs were administered to C57BL/6 J mice via tail-vein injection, followed by the establishment of a PDAC liver-metastasis model (n = 6 per group) (Fig. 2G). We observed that mice pretreated with EVs derived from the senescent group (S-EVs) developed liver metastases that grew more rapidly and exhibited a significantly higher number and burden of metastatic lesions compared with mice treated with EVs from the non-senescent group (N-EVs) (Fig. 2H–J). Consistently, the overall survival of mice in the S-EV group was significantly shorter than that of the N-EV group (Fig. 2K). Furthermore, we isolated EVs from KPC cells treated with low-dose AG in vitro and found that these senescent cell-derived EVs recapitulated the pro-metastatic phenotype observed in vivo (Supplementary Fig. 2C-K). To further validate these findings, we employed the senolytic agent ABT737 to eliminate senescent KPC cells and isolated their EVs for in vivo assessment (Fig. 2L). Previous studies have demonstrated the senolytic efficacy of ABT-737 in eliminating senescent pancreatic cancer cells [30]. Through SA-β-gal staining, we verified its capacity to eliminate senescent KPC cells induced by AG treatment (Fig. 2M, Supplementary Fig. 2L). We found that, compared to mice treated with EVs derived from the AG-induced senescence group, mice receiving EVs from the AG + ABT737 group exhibited significantly lower liver metastatic activity, tumor burden, and nodule counts (Fig. 2N-P). Additionally, these mice demonstrated a significantly prolonged survival time (Fig. 2Q). Taken together, these results indicated that EVs derived from NAC-induced senescent PDAC cells promoted liver metastasis.

### Senescent PDAC-released EVs reshaped the hepatic premetastatic niches

**Fig. 3:**
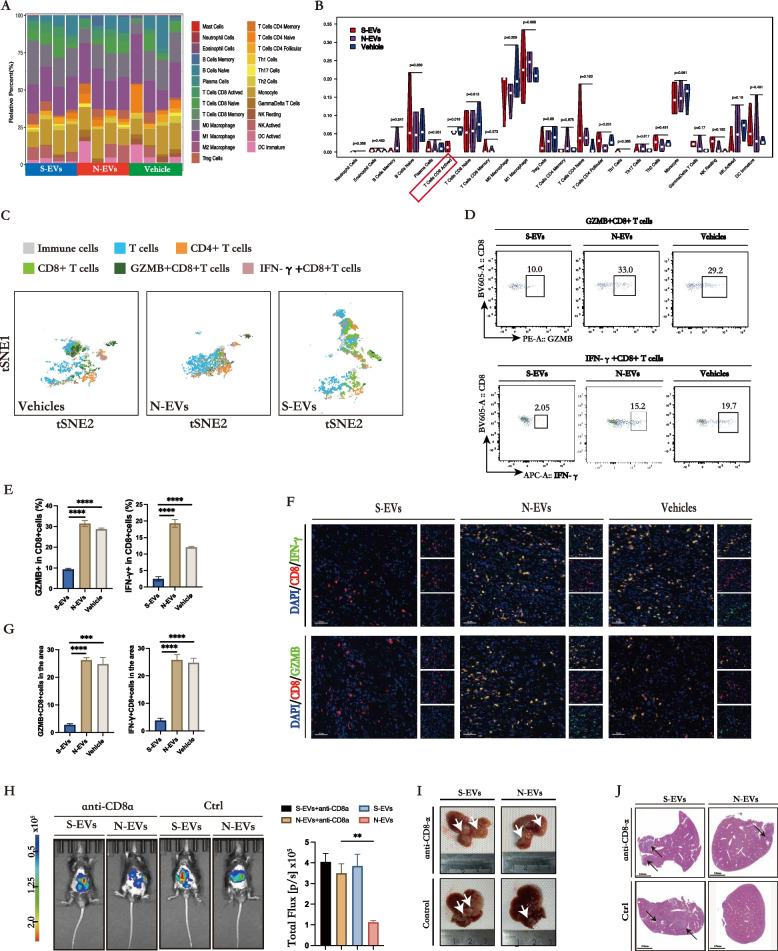
Senescent PDAC-released EVs reshaped the hepatic premetastatic niches. **A** Landscape of infiltrating immune cell populations in liver metastases, estimated by CIBERSORT analysis of bulk RNA-seq data. **B** Relative abundance of immune cell subsets in the S-EVs, N-EVs, and Vehicle groups. Note the significant reduction in activated CD8⁺ T cells in the S-EV group. **C** t-SNE visualization of hepatic immune cells analyzed by flow cytometry. **D**, **E** Flow cytometric quantification of cytotoxic activation markers GZMB and IFN-γ on infiltrating CD8⁺ T cells from liver metastases. **F**, **G** Representative multiplex immunohistochemistry (mIHC) images showing the spatial distribution of GZMB⁺CD8⁺ and IFN-γ⁺CD8⁺ T cells in liver metastatic tissues. CD8⁺ T cells are stained in red, GZMB or IFN-γ in green, and nuclei in blue (DAPI). **H** Representative in vivo bioluminescence images of liver metastases in mice treated with S-EVs or N-EVs following CD8⁺ T cell depletion using an anti-CD8α antibody (left), and quantification of the bioluminescence signals (right). **I**, **J** Representative macroscopic images and H&E staining of liver tissues showing metastatic burden in the indicated groups

We first evaluated the direct impact of EVs on KPC cells in vitro. Interestingly, S-EVs and N-EVs showed no significant differences in promoting cell proliferation, migration, or invasion (Supplementary Fig. 3A–C). Consequently, we hypothesized that the pro-metastatic effect of S-EVs relies on the modulation of the tumor microenvironment (TME). CIBERSORT analysis of bulk RNA-seq data from liver metastases identified CD4⁺/CD8⁺ T cells and macrophages as the dominant infiltrating immune populations (Fig. 3A), with a significantly reduced activated CD8⁺ T cell score in the S-EV group (Fig. 3B). Consistently, flow cytometry and t-SNE analysis (Supplementary Fig. 4A–C, Fig. 3C) showed a decrease in functional CD8⁺GZMB⁺ and CD8⁺IFN-γ⁺ T cells in S-EV-treated mice, indicating impaired cytotoxic activity (Fig. 3D–E). These results establish CD8⁺ T cells as a key target in the S-EV-induced microenvironment. Additionally, multiplex IHC staining of murine metastatic lesions revealed a significantly lower proportion of CD8⁺GZMB⁺ and CD8⁺IFN-γ⁺ cells in the S-EV group compared to the N-EV and vehicle groups (Fig. 3F–G). To elucidate the pivotal role of CD8⁺ T cells in S-EV-driven liver metastasis, we administered an anti-CD8α antibody (clone 2.43) to deplete CD8⁺ T cells in vivo. Following depletion, we observed no significant difference in survival rates or liver metastatic burden between the S-EV and N-EV groups (Fig. 3H-J). Additionally, we assessed the immunosuppressive status of the orthotopic pancreatic tumors using flow cytometry. The pancreatic tumor microenvironment exhibited no significant differences in the frequencies of GZMB⁺ or IFN-γ⁺ CD8⁺ T cells between the S-EV and N-EV groups (Supplementary Fig. 5A). Collectively, these results indicate that S-EVs facilitate the establishment of a liver-specific pro-metastatic niche by impairing the cytotoxicity of CD8⁺ T cells.

### S-EVs impaired CD8^+^ T cells anti-tumor immunity by promoting phosphatidylcholine accumulation in liver

**Fig. 4:**
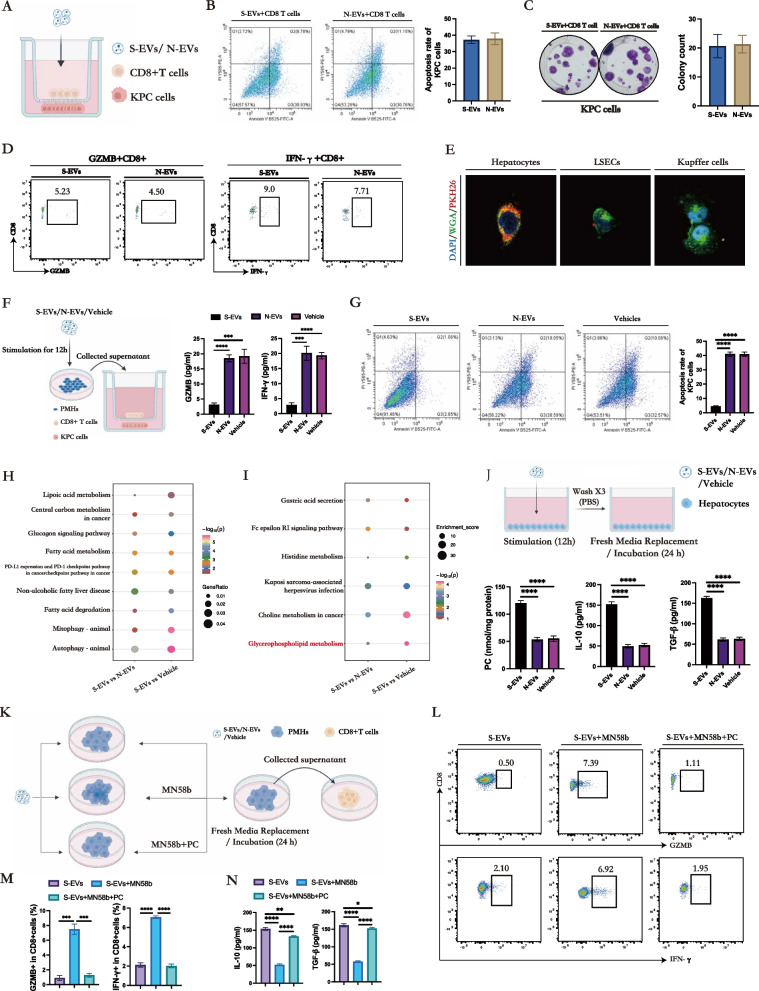
S-EVs impaired CD8.^+^ T cells anti-tumor immunity by promoting phosphatidylcholine accumulation in liver. **A** Schematic illustration of the co-culture system: CD8⁺ T cells were directly exposed to S-EVs, N-EVs, or vehicle prior to co-culture with KPC cells. **B**, **C** Assessment of direct effects of S-EVs on CD8⁺ T cell cytotoxicity. Apoptosis of KPC cells was measured by flow cytometry (**B**), and cell viability was visualized by crystal violet staining (**C**). **D** Flow cytometric analysis of GZMB and IFN-γ expression in CD8⁺ T cells after direct treatment with the indicated EVs. **E** Representative confocal microscopy images showing the uptake of PKH26-labeled S-EVs (red) by primary mouse hepatocytes (PMHs), liver sinusoidal endothelial cells (LSECs), and Kupffer cells. Nuclei were stained with DAPI (blue) and cell membranes with WGA (green). **F** Schematic workflow for evaluating the indirect effect of S-EV-educated hepatocytes on CD8⁺ T cells. Conditioned medium (CM) from PMHs treated with EVs was applied to the CD8⁺ T cell-KPC co-culture system. **G** Flow cytometric analysis of KPC cell apoptosis induced by CD8⁺ T cells in the presence of CM from PMHs treated with S-EVs, N-EVs, or vehicle. **H** KEGG pathway enrichment analysis of differentially expressed genes (DEGs) from RNA-seq data of liver metastases (S-EVs vs. N-EVs and S-EVs vs. Vehicle). **I** KEGG pathway enrichment analysis of differential metabolites identified in liver metastases, highlighting Glycerophospholipid metabolism as the top enriched pathway. **J** Quantification of intracellular PC levels in PMHs (left) and secretion of immunosuppressive cytokines (IL-10 and TGF-β) in the supernatant (right) following EV treatment. Data were analyzed by ELISA. **K** Quantification of intracellular PC levels in PMHs treated with MN58b (choline kinase inhibitor) to validate PC depletion efficacy. **L**-**N** Rescue experiments evaluating CD8⁺ T cell function. CD8⁺ T cell cytotoxic markers (GZMB/IFN-γ) (**L**, **M**) and immunosuppressive cytokines (IL-10/TGF-β) (N) and were assessed after co-culture with CM from S-EV-treated PMHs in the presence or absence of MN58b (PC depletion)

To investigate whether S-EVs directly affect CD8⁺ T cells, CD8⁺ T cells were co-cultured with KPC cells in the presence of S-EVs, N-EVs or vehicle (Fig. 4A). CD8⁺ T cells pretreated with S-EVs did not exhibit reduced cytotoxicity against tumor cells compared with those exposed to N-EVs (Fig. 4B–C). Consistently, GZMB and IFN-γ expression in CD8⁺ T cells did not differ significantly between the S-EV and N-EV groups (Fig. 4D). This suggests that the in vivo suppression of CD8⁺ T cell cytotoxicity by S-EVs involves other cell subpopulations in liver. We subsequently isolated three major hepatic cell populations—primary mouse hepatocytes (PMHs), liver sinusoidal endothelial cells (LSECs), and Kupffer cells—and co-cultured them with PKH26-labeled S-EVs for 24 h. PMHs exhibited significantly higher PKH26 fluorescence intensity than LSECs or Kupffer cells, indicating a superior uptake capacity for S-EVs. These data suggest that hepatocytes are the key mediators of S-EV-induced CD8⁺ T cell suppression (Fig. 4E). To evaluate the functional consequences of hepatocyte-derived soluble factors, KPC cells were incubated with conditioned medium (CM) from PMHs pre-treated with EVs prior to co-culture with CD8⁺ T cells. Quantitative analysis demonstrated that the CM from S-EV-stimulated PMHs significantly attenuated the cytotoxic potential and antitumor efficacy of CD8⁺ T cells (Fig. 4F-G). To investigate the underlying mechanism, we performed KEGG pathway enrichment analysis on the RNA-seq data from liver metastases (previously described in Fig. 3A). The analysis revealed a significant enrichment of metabolism-related pathways in the S-EVs group (Supplementary Fig. 6A–B; Fig. 4H). To further explore these metabolic alterations, we performed metabolomic profiling of the liver metastases. The results showed that differential metabolites were primarily enriched in Glycerophospholipid metabolism, with phosphatidylcholine (PC) displaying the most significant variation (Supplementary Fig. 6C; Fig. 4I). We highlighted the top five differentiating metabolites in the metabolomic profiling analysis, with PC (18:0/PGF1α) and PC (18:2/P-16:0) showing the most significant changes (Supplementary Fig. 6D). Following treatment with S-EVs, N-EVs, or Vehicle, we examined the metabolic and secretory profiles of PMH cells. S-EV-treated PMHs exhibited a distinct phenotype characterized by markedly increased intracellular PC levels and enhanced secretion of immunosuppressive cytokines (IL-10 and TGF-β) relative to controls (Fig. 4J). To validate the contribution of PC to this process, we utilized MN58b to deplete intracellular PC in S-EV-stimulated hepatocytes (Fig. 4K). In co-culture assays, PC depletion rescued the impaired cytolytic activity of CD8⁺ T cells, whereas exogenous PC supplementation inhibited this activity (Fig. 4L–M). Additionally, PC depletion reduced the levels of IL-10 and TGF-β in the hepatocyte supernatant, and the exogenous addition of PC restored their levels (Fig. 4N). These findings indicated that S-EVs dampen CD8⁺ T cell antitumor efficacy through the aberrant accumulation of intracellular PC.

### S-EV–derived hnRNPA1 stabilized AChE mRNA to promote PC accumulation

**Fig. 5:**
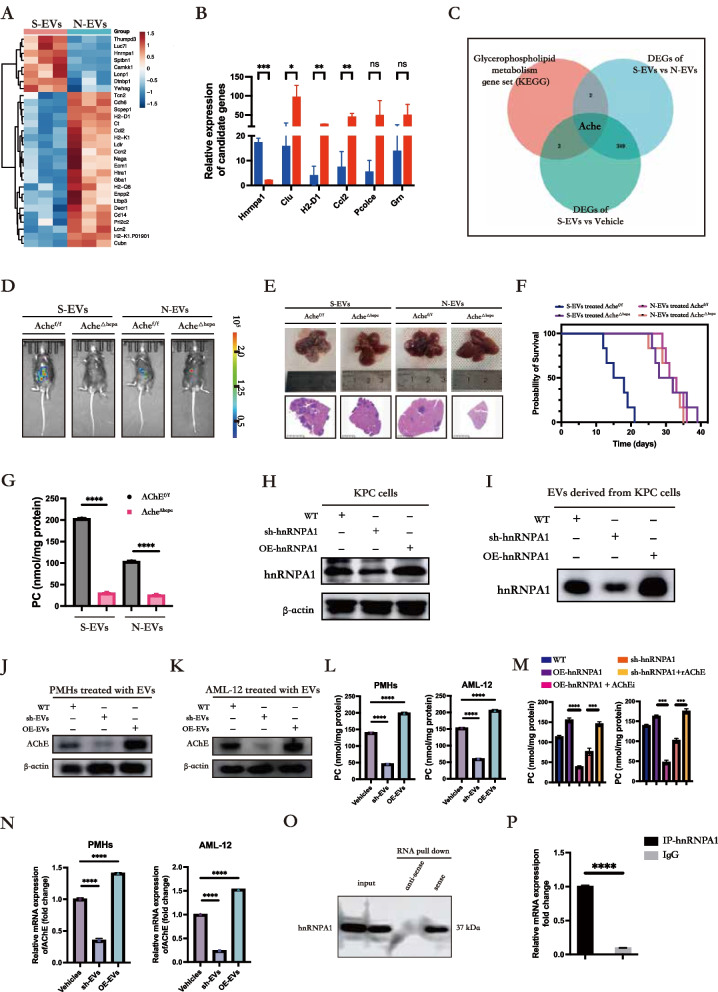
S-EV–derived hnRNPA1 stabilized AChE mRNA to promote PC accumulation. **A** Heatmap visualization of the top 30 differentially expressed proteins (DEPs) identified by proteomic analysis of S-EVs versus N-EVs. **B** Relative abundance of key candidate proteins (hnRNPA1, Clu, H2-D1, Ccl2, Pcolce, and Grn) in S-EVs and N-EVs. **C** Venn diagram identifying Ache as a potential target by intersecting DEGs from RNA-seq data (S-EVs vs. N-EVs; S-EVs vs. Vehicle) with the KEGG "Glycerophospholipid metabolism" gene set. **D** Representative in vivo bioluminescence images showing liver metastatic burden in AcheΔhepa and Achef/f mice pretreated with S-EVs or N-EVs (*n* = 6 per group). **E**, **F** Representative macroscopic images (**E**) and H&E staining (**F**) of liver tissues from the indicated mouse groups. (*n* = 6 per group). **G** Quantification of PC levels in liver metastatic tissues from the indicated groups. **H**, **I** Immunoblot analysis validating hnRNPA1 knockdown or overexpression efficacy in KPC cells (**H**) and their derived EVs (**I**). **J**, **K** Immunoblot analysis of AChE expression in PMHs (**J**) and AML-12 cells (**K**) treated with EVs derived from hnRNPA1-knockdown (sh-hnRNPA1) or -overexpressing (Flag-hnRNPA1) KPC cells. **L** ELISA quantification of PC levels in PMHs and AML-12 cells treated with the indicated EVs. **M** Rescue experiments analyzing PC levels in supernatants of PMHs and AML-12 cells. Cells were treated with hnRNPA1-modified EVs in the presence or absence of recombinant AChE (rAChE) or the AChE inhibitor. **N** RT-qPCR analysis of Ache mRNA levels in PMHs and AML-12 cells treated with the indicated EVs. **O** RNA pull-down assay confirming the direct physical interaction between biotinylated Ache mRNA and hnRNPA1 protein. **P** RNA immunoprecipitation (RIP) assay followed by RT-qPCR analysis showing the enrichment of Ache mRNA bound to hnRNPA1 protein

We further aimed to elucidate the mechanism of S-EVs in regulating PC accumulation of PMHs. Through proteomic analysis of EVs, we observed distinct differences in the protein cargo between S-EVs and N-EVs (Supplementary Fig. 7A). The top 30 differentially expressed proteins (DEPs) are shown in Fig. 5A. Subsequently, using leave-one-out cross-validation analysis, we identified six key proteins in S-EVs that are most strongly associated with liver metastasis: hnRNPA1, Clu, H2-D1, Ccl2, Pcolce, and Grn (Supplementary Fig. 7B-C). Notably, hnRNPA1 was identified as the most significantly upregulated component within S-EVs (Fig. 5B). To further elucidate the underlying molecular mechanisms, we performed an in-depth interrogation of our previously acquired RNA-Seq data from EVs treated liver metastatic tissues (refer to Fig. 3A). By intersecting the metabolic genes specifically differentially expressed in the S-EV group with the "Glycerophospholipid metabolism" gene set from the KEGG database, we identified AChE as a critical candidate. This analysis suggests that AChE may serve as a key regulator mediating S-EV-driven hepatic PC accumulation, thereby facilitating the formation of the hepatic PMNs (Fig. 5C). Notably, analysis of pancreatic tissues from mice treated with S-EVs or N-EVs revealed no significant difference in AChE expression (Supplementary Fig. 8A). This indicates that the S-EV-mediated upregulation of AChE is liver-specific. To validate the functional role of AChE in S-EV-mediated PDAC liver metastasis, we generated hepatocyte-specific Ache knockout mice (Ache^Δhepa^) (Supplementary Fig. 9A-B). Following pre-conditioning with S-EVs, Ache^Δhepa^ mice exhibited significantly reduced metastatic burden and tumor activity compared to the Ache^f/f^ mice, leading to markedly prolonged overall survival (Fig. 5D-F). Concomitantly, the hepatic PC accumulation typically induced by S-EVs was significantly abrogated in the Ache^Δhepa^ mice (Fig. 5G). To validate the functional role of hnRNPA1 in EVs, we established stable KPC cell lines with hnRNPA1 knockdown or overexpression (Fig. 5H). EVs were isolated from these lines, and the differential abundance of hnRNPA1 was verified via immunoblotting (Fig. 5I). We then co-cultured these engineered EVs with both mouse primary hepatocytes and the normal hepatocyte cell line AML-12. The results demonstrated that EVs derived from hnRNPA1-knockdown cells significantly reduced intracellular AChE expression and PC content in hepatocytes. Conversely, EVs from hnRNPA1-overexpressing cells markedly upregulated AChE expression and promoted PC accumulation (Fig. 5J-L). Further investigation revealed that manipulating AChE activity could reverse the regulatory effects of EVs derived hnRNPA1 on hepatic PC accumulation. Specifically, the decline in PC levels caused by hnRNPA1-knockdown EVs was significantly rescued by the exogenous addition of recombinant mouse AChE protein. Conversely, treatment with the AChE inhibitor pyridostigmine effectively abrogated the PC accumulation induced by hnRNPA1-overexpressing EVs (Fig. 5M). Given the canonical role of hnRNPA1 as an RNA-binding protein we hypothesized that it modulates AChE expression at the post-transcriptional level. Indeed, we observed that hnRNPA1-enriched EVs significantly upregulated AChE mRNA levels in both PMHs and AML-12 cells (Fig. 5N). Leveraging AlphaFold 3 for structural modeling, we predicted a high-confidence interaction interface between hnRNPA1 and AChE mRNA (ipTM + pTM > 1) (Supplementary Fig. 9C). Corroborating this prediction, RNA pull-down and RNA immunoprecipitation assays confirmed a direct physical association between hnRNPA1 and the AChE transcript. Mechanistically, this binding interaction was found to enhance the stability of AChE mRNA, thereby elevating its expression (Fig. 5O-P). Collectively, these results demonstrated that hnRNPA1 delivered by S-EVs stabilized AChE mRNA, leading to increased PC accumulation in liver.

### Inhibition of AChE with pyridostigmine restored CD8⁺ T cell anti-tumor immunity and suppressed PDAC liver metastasis

**Fig. 6:**
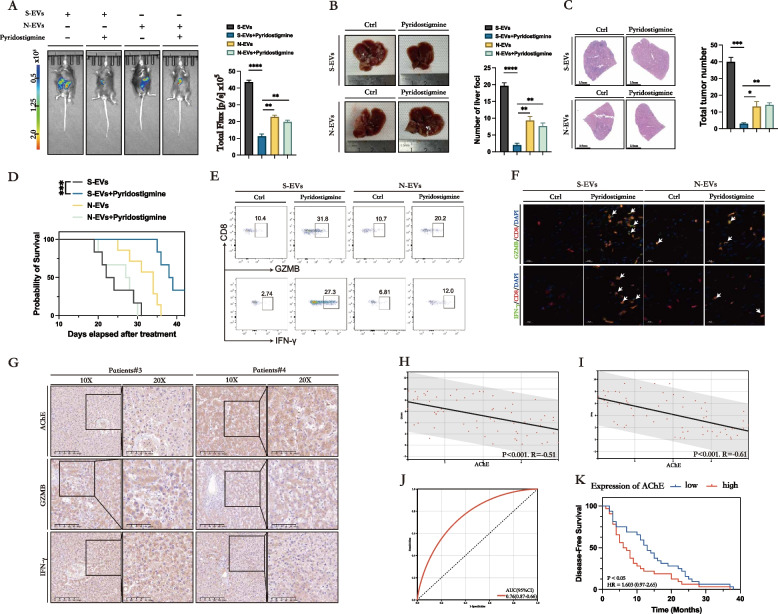
Inhibition of AChE with pyridostigmine restored CD8⁺ T cell anti-tumor immunity and suppressed PDAC liver metastasis. **A** Representative in vivo bioluminescence images monitoring liver metastasis progression in mice treated with pyridostigmine followed by S-EV or N-EV preconditioning (left), and quantification of the bioluminescence signals (right). *n* = 6 per group. **B**, **C** Assessment of liver metastatic burden (*n* = 6 per group). **B** Representative macroscopic images of liver tissues (left) and quantification of nodule numbers (right). **C** Representative H&E staining of liver sections (left) and quantification of tumor area (right). **D** Kaplan–Meier survival curves showing overall survival (OS) of tumor-bearing mice in the indicated groups (*n* = 6 per group). **E** Flow cytometric analysis and quantification of GZMB and IFN-γ expression in infiltrating hepatic CD8⁺ T cells from the indicated groups. **F** Representative multiplex immunohistochemistry (mIHC) images showing the infiltration of GZMB⁺CD8⁺ and IFN-γ⁺CD8⁺ T cells in liver metastatic tissues. **G** Representative IHC staining of AChE, GZMB and IFN-γ in PDAC specimens from patients treated with NAC. **H**, **I** Pearson correlation analysis between AChE expression levels and the cytotoxic markers GZMB (**H**) and IFN-γ (**I**) in liver metastasis specimens from NAC-treated PDAC patients. **J** ROC curve analysis evaluating the predictive value of serum AChE levels for early postoperative liver metastasis in PDAC patients receiving NAC. The area under the curve (AUC) is 0.76 (95% CI: 0.66–0.87). **K** Kaplan–Meier analysis of disease-free survival (DFS) in NAC-treated PDAC patients stratified by serum AChE levels (high vs. low). High serum AChE expression is associated with poor prognosis (HR = 1.603, *P* < 0.05)

To investigate the therapeutic potential of the AChE inhibitor pyridostigmine against PDAC liver metastasis, we established a liver metastasis model in mice pretreated with S-EVs or N-EVs, followed by intragastric administration of pyridostigmine (15 mg/kg every 3 days). In vivo results demonstrated that pyridostigmine treatment significantly suppressed S-EV-induced metastatic growth and reduced tumor burden, while markedly prolonging survival compared to the control group (Fig. 6A–D). Mechanistically, flow cytometry analysis revealed enhanced cytotoxicity of CD8⁺ T cells in the pyridostigmine-treated group (Fig. 6E), and mIHC further confirmed a significant increase in the infiltration of GZMB⁺ and IFN-γ⁺ CD8⁺ T cells within liver tissues (Fig. 6F), suggesting that pyridostigmine activates anti-tumor immunity to potentially augment neoadjuvant chemotherapy (NAC) efficacy. To validate the clinical relevance of these findings, we analyzed paired liver metastasis tissues and blood samples from NAC-treated PDAC patients undergoing re-operation for liver metastasis (Fig. 6G). Correlation analysis showed that hepatic AChE expression was significantly negatively correlated with the cytotoxic markers GZMB (R = 0.54, *P* < 0.001) and IFN-γ (R = 0.61, *P* < 0.001) (Fig. 6H–I). Furthermore, ELISA quantification indicated a strong positive correlation between serum AChE levels and hepatic AChE expression (R = 0.90, *P* < 0.001) (Supplementary Fig. 10A). Notably, ROC curve analysis demonstrated that serum AChE levels exhibited predictive value for early postoperative liver metastasis recurrence (AUC = 0.76; 95% CI: 0.66–0.87) (Fig. 6J), and survival analysis confirmed that high serum AChE expression was associated with significantly shorter disease-free survival (HR = 1.603, *P* < 0.05) (Fig. 6K), indicating that serum AChE may serve as a potential biomarker for predicting early liver metastasis in NAC-treated PDAC patients.

## Discussion

**Fig. 7:**
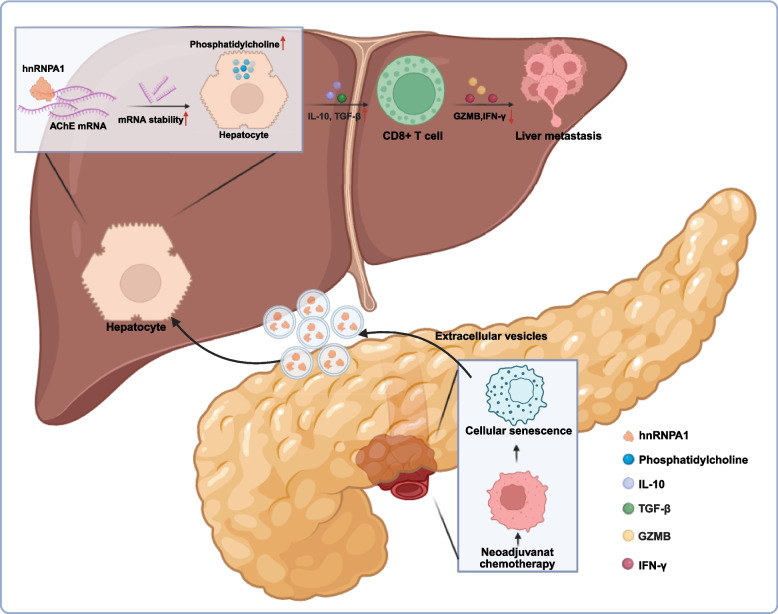
Schematic illustration of S-EVs-mediated hepatic PMN reprogramming in PDAC liver metastasis. Chemotherapy-induced senescent PDAC cells release S-EVs that reshape hepatic PMNs through AChE-dependent PC accumulation, subsequently impairing CD8 + T cell cytotoxicity and ultimately promoting PDAC liver metastasis

Although NAC has improved surgical resection rates and long-term survival in PDAC patients, nearly one-third of patients develop liver metastases within 6 months postoperatively [31, 32]. This clinical challenge underscores the urgent need to elucidate the underlying mechanisms and identify novel therapeutic strategies. Here, we demonstrate that NAC-induced senescence in primary PDAC tumors is significantly associated with early liver metastasis and poor prognosis. Mechanistically, we reveal that S-EVs facilitate the formation of a hepatic PMNs by suppressing CD8⁺ T cell cytotoxicity through AChE-dependent accumulation of phosphatidylcholine (Fig. 7). Consequently, our findings suggest that pharmacological inhibition of AChE with pyridostigmine represents a potential therapeutic strategy to mitigate liver metastasis in PDAC patients undergoing NAC.

Our clinical cohort analysis revealed that PDAC patients with pronounced primary tumor senescence after neoadjuvant chemotherapy are at a significantly higher risk for postoperative liver metastasis. We observed a markedly higher level of senescence in the primary PDAC lesions of the liver metastasis group compared to the non-metastasis group. Furthermore, elevated expression of senescence-associated markers (p16/p21/p53) in the primary tumor correlated with shorter OS and DFS in NAC-treated PDAC patients. This aligns with findings by Zhou et al., who demonstrated that heterogeneous expression of senescence markers (p16 and p21) following NAC predicts early tumor recurrence in colorectal cancer [20]. Taken together, these findings highlight the heterogeneity in liver metastasis risk among patients receiving neoadjuvant chemotherapy. This provides a critical rationale for clinical stratification, enabling the early identification of individuals at an elevated risk for post-NAC metastasis.

In the present study, we identified NAC-mediated tumor senescence as a pivotal driver of liver metastasis and a determinant of poor prognosis in PDAC patients. Cellular senescence is characterized by stable cell cycle arrest triggered by diverse stressors, including oncogene activation, DNA damage, and anti-cancer therapies [18, 33]. The role of senescence in cancer progression remains a subject of intense debate due to its complex, context-dependent nature. The previous studies using genetically engineered mouse models have established senescence as a tumor-suppressive mechanism that restricts malignant expansion and recruits immune clearance [34, 35]. Conversely, emerging evidence suggests that therapy-induced senescence inadvertently fuels tumor recurrence and metastasis [36]. This dichotomy is largely attributed to the senescence-associated secretory phenotype, through which senescent cells remodel the tumor microenvironment via the release of soluble factors and EVs, thereby paradoxically enhancing tumor cell survival and migration [37, 38].

NAC efficacy is compromised not only by the development of drug resistance but also, more importantly, by NAC-induced alterations in the tumor microenvironment. At present, multiple studies have shown that NAC can modify the tumor immune microenvironment in PDAC [39]. For instance, the frequency of conventional CD4⁺ T cells is increased and the proportion of Tregs is reduced in the pancreatic tumor microenvironment after neoadjuvant treatment [40]. Previous work has also indicated that, following NAC, T-cell infiltration increases, whereas macrophage and granulocyte infiltration decreases [41]. In addition, NAC has been shown to upregulate CD36 on effector T cells, and targeting CD36 can not only restore anti-tumor immunity but also suppress chemoresistance [42]. However, these studies do not clarify the mechanisms driving liver metastasis after NAC. In this study, our in vivo and in vitro experiments demonstrate that NAC-induced senescent PDAC cells release S-EVs, which orchestrate immunosuppressive PMNs to facilitate liver metastasis. Flow cytometry demonstrated that S-EVs promoted the formation of immunosuppressive PMNs by suppressing CD8⁺T cell cytotoxicity, thereby facilitating liver metastasis. Mechanistically, S-EVs upregulated AChE in the liver, promoting hepatic PC accumulation and, in turn, increasing IL-10 and TGF-β levels, which suppress CD8⁺ T-cell cytotoxicity [43, 44]. By inhibiting PC synthesis and adding exogenous PC to hepatocyte culture media, we demonstrated that S-EVs regulate CD8⁺ T cell anti-tumor immunity via AChE-dependent phospholipid metabolic reprogramming. Multiple studies have shown that impaired CD8⁺ T cell anti-tumor function can promote liver metastasis in pancreatic cancer. Functional impairment of cytotoxic T cells in PDAC liver metastases–associated regions suggests that an immunosuppressive microenvironment is an important prerequisite for metastatic spread [45]. Additionally, metastasis-associated fibroblasts are able to suppress CD8⁺ T-cell cytotoxicity through cytokine secretion [46]. Our study highlighted the role of AChE-driven phospholipid metabolic reprogramming in hepatocytes in regulating CD8⁺ T cell cytotoxicity, suggesting a potential combination strategy for PDAC patients receiving NAC.

Furthermore, we investigated the mechanism of S-EVs upregulating hepatic AChE. Our previous work showed that EVs released from untreated PDAC cells carried CD44v6/C1QBP complex and shaped hepatic PMNs by promoting liver fibrosis [21]. However, NAC has been shown to alter the molecular biological features of tumors [47], which may in turn change the cargo of tumor-derived EVs. We found that EVs from NAC-induced senescent PDAC cells displayed a markedly different proteomic profile compared with EVs from untreated PDAC cells, supporting a distinct role of S-EVs in PDAC liver metastasis. Using proteomic analysis combined with leave-one-out screening, we identified hnRNPA1 as the key S-EV protein regulating hepatic AChE. hnRNPA1 is a member of the RNA-binding protein family, and multiple studies have shown that hnRNPA1 can bind to mRNA, increase its stability, and thereby enhance its expression [48–51]. RIP and RNA pull-down assays further confirmed that hnRNPA1 derived from S-EVs binds to and stabilizes AChE mRNA, promoting AChE expression. These findings highlight the important role of S-EV–derived hnRNPA1 in PDAC liver metastasis and indicate a key direction for our future studies.

The present study showed that targeting AChE potentially remodel the hepatic PMNs in PDAC patients with NAC-induced senescence in primary tumors, characterized by enhanced cytotoxicity of CD8⁺ T cells. AChE inhibition markedly suppressed liver metastasis and prolonged survival in mice. These evidences indicated that AChE blockade reversed the immunosuppressive microenvironment shaped by S-EVs and reduced subsequent liver metastasis. The AChE inhibitor pyridostigmine has already demonstrated favorable safety and clinical efficacy in routine use for myasthenia gravis, yet its role in PDAC has not been reported. Our data supported the repurposing of pyridostigmine in PDAC, in combination with anti–PD-1, to enhance the therapeutic benefit of NAC. In preclinical models, pyridostigmine increased sensitivity to anti–PD-1, resulting in more pronounced metastases control and improved OS. These results suggested that a pyridostigmine-based PMN reprogramming strategy could strengthen the impact of NAC in PDAC and have potential application in immunotherapy for in PDAC liver metastases.

In summary, this study reveals the critical role of NAC-induced senescent primary tumors in regulating immunosuppressive hepatic PMNs of PDAC. S-EVs deliver hnRNPA1, which impairs CD8⁺ T-cell function through AChE-dependent phospholipid metabolic reprogramming. Targeting AChE reverses NAC-induced suppressive PMNs, and pyridostigmine has the potential to suppress liver metastasis in PDAC. In addition, PMN reshaping with pyridostigmine might be a promising strategy to enhance the efficacy of NAC.

## Conclusions

Collectively, our findings demonstrate that S-EVs derived from chemotherapy-induced senescent PDAC cells remodel the hepatic immune microenvironment by driving AChE-dependent PC accumulation. This metabolic reprogramming of PMNs suppresses CD8⁺ T cell cytotoxicity, thereby facilitating PDAC liver metastasis. Consequently, pharmacological inhibition of AChE with pyridostigmine emerges as a viable therapeutic strategy to disrupt this immunosuppressive axis, potentially preventing metastatic progression and enhancing the efficacy of NAC.

## Supplementary Information

Supplementary Material 1.

## Data Availability

The datasets used or analysed during the current study are available from the corresponding author on reasonable request.

## Abbreviations

NAC: Neoadjuvant chemotherapy
PDAC: Pancreatic ductal adenocarcinoma
EVs: Extracellular vesicles
PMNs: Pre-metastatic niches
PC: Phosphatidylcholine
AChE: Acetylcholinesterase
hnRNPA1: Heterogeneous nuclear ribonucleoprotein A1
TEM: Transmission electron microscope
NTA: Nano-particle Tracking Analysis
AG: Gemcitabine plus nab-paclitaxel
GZMB: Granzyme B
IFN-γ: Interferon-gamma
