# Supplementary material for: Extracellular vesicles from chemotherapy-induced senescent tumor cells reprogram hepatic phospholipid metabolism to promote pancreatic cancer liver metastasis
Source: J Exp Clin Cancer Res. 2026 May 22;45:163. doi: 10.1186/s13046-026-03741-3 (PMC13374290; doi:10.1186/s13046-026-03741-3)
Supplement: Supplementary file 1 — Supplementary Material 1. [file 13046_2026_3741_MOESM1_ESM.docx]

**Materials and Methods**

**RNA-seq**

Libraries for RNA-Seq were prepared with VAHTS Universal V10 RNA-seq Library Prep Kit (Premixed Version). The workflow consists of mRNA enrichment, cDNA generation, and end repair to generate blunt ends, A-tailing, adaptor ligation and PCR amplification. Different adaptors were used for multiplexing samples in one lane. Sequencing was performed on Illumina Novaseq 6000 and 150 bp paired-end reads were generated. The clean reads were mapped to the reference genome using HISAT2. FPKM of each gene was calculated and the read counts of each gene were obtained by HTSeq-count. PCA analysis were performed using R (v3.2.0) to evaluate the biological duplication of samples.

**Western blot**

Cells underwent lysis, and the protein concentration was assessed through the BCA method. Proteins were isolated on 10% or 12% SDS-PAGE gels and then transferred to NC membranes using the Mini-Trans Blot electrophoretic transfer system. After washing and blocking, the membranes were incubated with the primary antibody at 4°C overnight. Following the washing of the membranes, they were treated with a secondary antibody linked to HRP, and detection was carried out using the ECL technique. Information of the antibodies are listed in Supplementary Table 1.

**Immunohistochemistry**

The paraffin sections were first deparaffinized using two xylene changes and then rehydrated with ethanol in decreasing concentrations. Antibody activity was restored using sodium citrate (0.1 M, pH 6.0) in a microwave for 20 minutes. A 3% hydrogen peroxide solution was used to inactivate endogenous peroxidase at room temperature for 10 minutes. After being washed three times in PBS, the tissue sections were blocked for 35 minutes at room temperature. The sections were exposed to primary antibodies overnight at 4 °C and then washed in PBS four times for 15 minutes each. The sections were incubated with the appropriate secondary antibodies for 35 minutes at room temperature, followed by four 15-minute washes in PBS. DAB staining and hematoxylin counterstaining were conducted, and images were captured with an Olympus microscope (Olympus, Tokyo, Japan). Duplicate sections were used as a negative control by immunostaining them with PBS in place of primary antibodies. Information of the antibodies are listed in Supplementary Table 1.

**Multiplex immunohistochemistry staining**

Multiplex immunohistochemistry staining was performed using Tyramide Signal Amplification (TSA) to enable sequential staining on single FFPE sections. Following antigen retrieval in Tris-EDTA (pH 9.0), sections underwent cyclic rounds of staining: overnight incubation with primary antibodies, HRP-conjugated secondary antibody labeling, tyramide-fluorophore deposition (Try-488, Try-Cy3, Try-Cy5), and heat-induced antibody stripping. Human PDAC sections were stained for p16, p21, p53, CK-19, CD45, and α-SMA, while mouse liver tissues were stained for CD8, GZMB, and IFN-γ. Nuclei were counterstained with DAPI. Information of the antibodies are listed in Supplementary Table 1.

***In vitro* senescence and senolytic interventions**

To induce cellular senescence *in vitro*, KPC cells were subjected to a 3-day continuous treatment with a low-dose combination of gemcitabine (10 nM) and nab-paclitaxel (5 nM). To evaluate senolytic efficacy, the AG-induced senescent KPC cells were exposed to 5 μM ABT-737 for 24 hours. Following drug withdrawal and medium replacement, cellular senescence was assessed via SA-β-gal staining.

**Flow cytometry analysis**

To prepare a single-cell suspension for *in vivo* flow cytometry, fresh mouse liver tissue of suitable size was dissected and mechanically separated using sterile ophthalmic scissors. Following this, the liver fragments were incubated at 37°C in a serum-free RPMI 1640 medium, which was supplemented with DNase I (0.1 mg/mL; Solarbio), collagenase I (1 mg/mL; Sigma-Aldrich), collagenase II (1 mg/mL; Sigma-Aldrich), and collagenase IV (1 mg/mL; Sigma-Aldrich) for 30 min with continuous stirring. The suspension of single cells was filtered using a 70 µm cell strainer (Miltenyi). Next, the red blood cells within the liver tissue were lysed for 5 minutes with a red blood cell lysis buffer (Miltenyi) at room temperature. The cells were lysed and then spun at 400 g for 5 minutes at 4°C, after which the reaction was halted by adding RPMI 1640 medium with 5% fetal bovine serum (FBS). For *in vitro* flow cytometry, immune cells were gathered and rinsed three times with PBS prior to being transferred to centrifuge tubes. Cells were washed with PBS and stained with BD Horizon Fixable Viability Stain 510 (BD Biosciences) at a 1:1,000 dilution in PBS for 15 minutes at 4°C. For surface staining, cells were incubated with fluorescently labeled antibodies targeting surface proteins in a staining buffer at 4°C for 30 minutes. For intracellular staining, such as interferon-gamma (IFN-γ), and granzyme B (GZMB), cells were fixed and permeabilized after surface staining. Use the Fix/Perm Buffer (BD, 562574) and Perm/Wash Buffer (BD, 562574) according to the manufacturer's guidelines to fix and permeabilize the cells. Once fixation and permeabilization are complete, expose the samples to the suitable antibodies at 4°C for 30 minutes. Data acquisition for flow cytometry was executed using a CytoFLEX flow cytometer (Beckman Coulter), while FlowJo software (version 10.8.1, TreeStar) was used for data analysis. Information of the antibodies are listed in Supplementary Table 1.

***In vivo* EVs administration and tissue analysis**

To investigate the in vivo effects of EVs on both the local pancreatic tissue and the tumor microenvironment, C57BL/6 mice were utilized and assigned to either normal tissue evaluation or the orthotopic tumor model. S-EVs or N-EVs were systemically administered to mice via the tail vein. Two week later，pancreatic tissues were rapidly excised, homogenized, and processed for protein extraction to conduct Western blot analysis. For the orthotopic tumor model, mice were anesthetized, and KPC cells suspended in an equal volume of PBS and Matrigel were carefully injected into the tail of the pancreas. All mice were euthanized for tissue collection. The orthotopic pancreatic tumor tissues were harvested, mechanically minced, and enzymatically digested into single-cell suspensions for immune cell profiling via flow cytometry.

**Extracellular vesicle proteomic analysis**

Peptides derived from EVs were prepared via reduction, alkylation, and trypsin digestion, followed by C18 desalting. Liquid chromatography-tandem mass spectrometry (LC-MS/MS) was performed using an Orbitrap Astral Mass Spectrometer (Thermo Fisher Scientific). The instrument operated in Data-Independent Acquisition (DIA) mode to maximize proteome coverage and quantification accuracy. Full MS scans were acquired in the Orbitrap analyzer with a resolution of 240,000 (at 200 m/z), while high-speed MS/MS scans were acquired in the Astral analyzer using Higher-energy Collisional Dissociation (HCD) fragmentation. Raw data were processed using DIA-NN software with a library-free strategy, searching against the Mus musculus UniProt database. The False Discovery Rate (FDR) was set at 1% for both precursor and protein levels. To identify senescence-associated EV proteins, we applied a leave-one-out (LOO) strategy: in each iteration, one biological replicate was omitted and differential expression between S-EVs and N-EVs was recalculated. Proteins consistently altered across all LOO iterations were defined as robust candidates. These LOO-filtered proteins were further evaluated by fold-change ranking and pathway enrichment to nominate key effectors.

**LC-MS/MS Analysis**

The metabolomic data analysis was performed by Shanghai OE Biotech Co., Ltd. (Shanghai, China). An ACQUITY UPLC I-Class plus（Waters Corporation，Milford, USA） fitted with Q-Exactive mass spectrometer equipped with heated electrospray ionization (ESI) source (Thermo Fisher Scientific, Waltham, MA, USA) was used to analyze the metabolic profiling in both ESI positive and ESI negative ion modes. An ACQUITY UPLC HSS T3 column (1.8 μm, 2.1 × 100 mm) were employed in both positive and negative modes. The binary gradient elution system consisted of (A) water (containing 0.1 % formic acid, v/v) and (B) acetonitrile and separation was achieved using the following gradient: 0min, 5% B; 2min, 5% B; 4min, 30% B; 8min, 50% B; 10min, 80% B; 14min, 100% B; 15 min, 100% B; 15.1 min, 5% and 16 min, 5%B. The flow rate was 0.35 mL/min and column temperature was 45℃. All the samples were kept at 10℃ during the analysis. The mass range was from m/z 70 to 1050. The resolution was set at 60000 for the full MS scans and 15000 for HCD MS/MS scans. The Collision energy was set at 10, 20 and 40 eV. The mass spectrometer operated as follows: spray voltage, 3800 V (+) and 3200 V (−); sheath gas flow rate, 35 arbitrary units; auxiliary gas flow rate, 8 arbitrary units; capillary temperature, 320°C; Aux gas heater temperature, 350°C; S-lens RF level, 50.

**RNA pull-down assay**

RNA pull-down was performed using the Pierce™ Magnetic RNA–Protein Pull-Down Kit (Thermo Fisher Scientific) according to the manufacturer’s instructions. Briefly, biotinylated AChE mRNA (sense) and its antisense control were generated using the 3'-end biotinylation system provided in the kit and immobilized on streptavidin magnetic beads. Bead-bound RNA was equilibrated in RNA–protein binding buffer and incubated with lysates from primary mouse hepatocytes under native conditions. After stringent washing, RNA–protein complexes were eluted with SDS sample buffer and analysed by western blotting. Enrichment of hnRNPA1 in pull-downs with biotinylated AChE mRNA (sense) compared with the antisense probe confirmed the specific association between hnRNPA1 and AChE mRNA. RNA pull-down assay was performed with commercial kits (Supplementary Table 2) according to the manufacturer’s instructions.

**RNA immunoprecipitation (RIP)**

RNA–protein interactions between hnRNPA1 and AChE mRNA were examined using the PureBinding® RNA Immunoprecipitation Kit (Geneseed, Cat. P0101) according to the manufacturer’s protocol. Briefly, hepatocytes were lysed in RIP lysis buffer supplemented with RNase and protease inhibitors, and clarified lysates were incubated with protein A/G magnetic beads pre-bound to anti-hnRNPA1 antibody or control rabbit IgG. After extensive washing, RNA–protein complexes were eluted from the beads and RNA was purified on spin columns, followed by reverse transcription. Enrichment of AChE mRNA in hnRNPA1 RIP versus IgG control was quantified by RT–qPCR using gene-specific primers. RIP was performed with commercial kits (Supplementary Table 2) according to the manufacturer’s instructions.

**Real-Time PCR**

The total RNA was isolated using a TRIzol reagent (Invitrogen, Carlsbad, CA, USA) according to the manufacturer's instructions. Real-time PCR was performed using a PrimeScript RT Reagent Kit (Perfect Real Time) (Takara, USA) and TB Green probes (Takara, USA) according to the manufacturer's protocols. All measurements were performed in triplicate. β-actin was used as a reference gene to normalize gene expression. The values for relative quantification were calculated using the 2^−ΔΔCt^ method after the threshold cycle.

**Supplemental Table 1. Antibodies used for Western blotting,** **Immunohistochemistry, Flow cytometry, RNA immunoprecipitation, and Immunofluorescence**

| **Name** | **Cat#** | **Company** |
| --- | --- | --- |
| CDKN2A/P16-INK4A  Recombinant mAb | 81373-10-RR | Proteintech |
| Anti-CDKN2A/p16INK4a  antibody | ab211542 | Abcam |
| p21 Waf1/Cip1 (12D1)  Rabbit mAb | 2947T | Cell Signaling Technology |
| Anti-p53 antibody | ab26 | Abcam |
| Anti-Cytokeratin 19 antibody | ab52625 | Abcam |
| anti-CD45 antibody | ab40763 | Abcam |
| Alpha smooth muscle actin  antibody | 14395-1-AP | Proteintech |
| [Anti-CD63 antibody](https://www.abcam.com/en-us/products/primary-antibodies/cd63-antibody-epr21151-ab217345) | ab217345 | Abcam |
| Anti-TSG101 antibody | ab125011 | Abcam |
| Anti-Calnexin antibody | ab22595 | Abcam |
| Anti-Albumin antibody | ab207327 | Abcam |
| Anti-Granzyme B antibody | ab255598 | Abcam |
| IFN gamma Polyclonal  Antibody | PA5-95560 | ThermoFisher Scientific |
| Anti-CD8 alpha antibody | ab237709 | Abcam |
| Anti-CD9 alpha antibody | ab217344 | Abcam |
| Anti-Acetylcholinesterase  antibody | ab97299 | Abcam |
| Anti-hnRNP A1 antibody | ab137780 | Abcam |
| BD Pharmingen™ APC-Cy™7 Rat  Anti-Mouse CD45 | 557659 | BD Pharmingen |
| BD Horizon™ RB705 Hamster  Anti-Mouse CD3e | 570560 | BD Pharmingen |
| BD Pharmingen™ FITC Rat  Anti-Mouse CD4 | 557307 | BD Pharmingen |
| BD Pharmingen™ PE-Cy™7 Rat  Anti-Mouse CD8a | 552877 | BD Pharmingen |
| BD Pharmingen™ PE Mouse  Anti-Mouse NK-1.1 | 557391 | BD Pharmingen |
| BD Horizon™ BV421 Rat  Anti-Mouse CD25 | 564370 | BD Pharmingen |
| BD Pharmingen™ Alexa Fluor® 647 Rat  anti-Mouse Foxp3 | 560401 | BD Pharmingen |
| BD Horizon™ Fixable  Viability Stain 510 | 564406 | BD Pharmingen |
| BD Pharmingen™ FITC Rat  Anti-CD11b | 557396 | BD Pharmingen |
| BD Horizon™ BV421 Rat  Anti-Mouse F4/80 | 565411 | BD Pharmingen |
| BD Pharmingen™ PE-Cy™7 Rat  Anti-Mouse CD86 | 560582 | BD Pharmingen |
| BD Pharmingen™ Alexa Fluor® 647 Rat  Anti-Mouse CD206 | 565250 | BD Pharmingen |
| BD Pharmingen™ PerCP-Cy™5.5 Rat  Anti-Mouse Ly-6G | 560602 | BD Pharmingen |
| BD Pharmingen™ PE Rat Anti-Mouse  Ly-6G and Ly-6C | 553128 | BD Pharmingen |

**Supplemental Table 2. Assay kits used for the measurements of phosphatidylcholine, IL-10, TGF-β and β-galactosidase, RNA-pull down, and RIP**

| **Name** | **Cat#** | **Company** |
| --- | --- | --- |
| Phosphatidylcholine | Ab83377 | Abcam |
| Mouse IL-10 ELISA Kit | ab255729 | Abcam |
| Human/Mouse TGF beta-1 Uncoated ELISA Kit | 88-8350-88 | ThermoFisher Scientific |
| Senescence-Associated β-Galactosidase(SA-β-Gal)Stain Kit | G1073-100T | Servicebio |
| PureBinding® RNA-Protein pull-down Kit | P0202 | GENESEED |
| PureBinding® RNA Immunoprecipitation Kit | P0102 | GENESEED |

**Supplementary Figures**

**
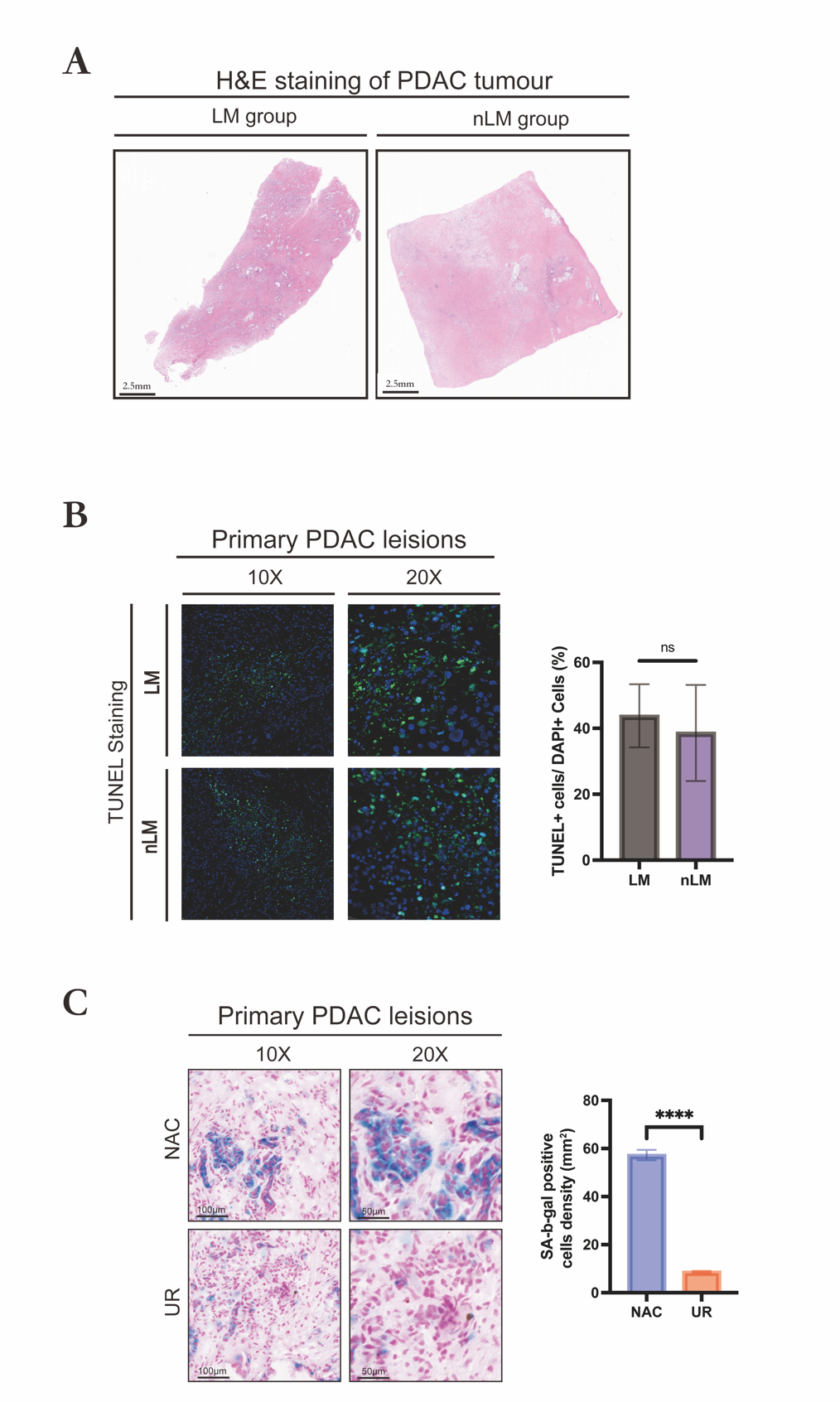
**

**Supplementary Figure.1 Histopathological characteristics and apoptotic status of primary PDAC tumors following neoadjuvant chemotherapy.**

(A) Representative H&E staining of primary tumors in PDCA patients treated with NAC.

(B) Representative TUNEL staining of primary tumors (left panel) and quantification of TUNEL-positive cells (right panel) in PDCA patients treated with NAC.

(C) Representative SA-β-gal staining of primary PDAC lesions (left panel) and quantification of SA-β-gal-positive cells (right panel) in PDCA patients with NAC or upfront resection (UR).

**
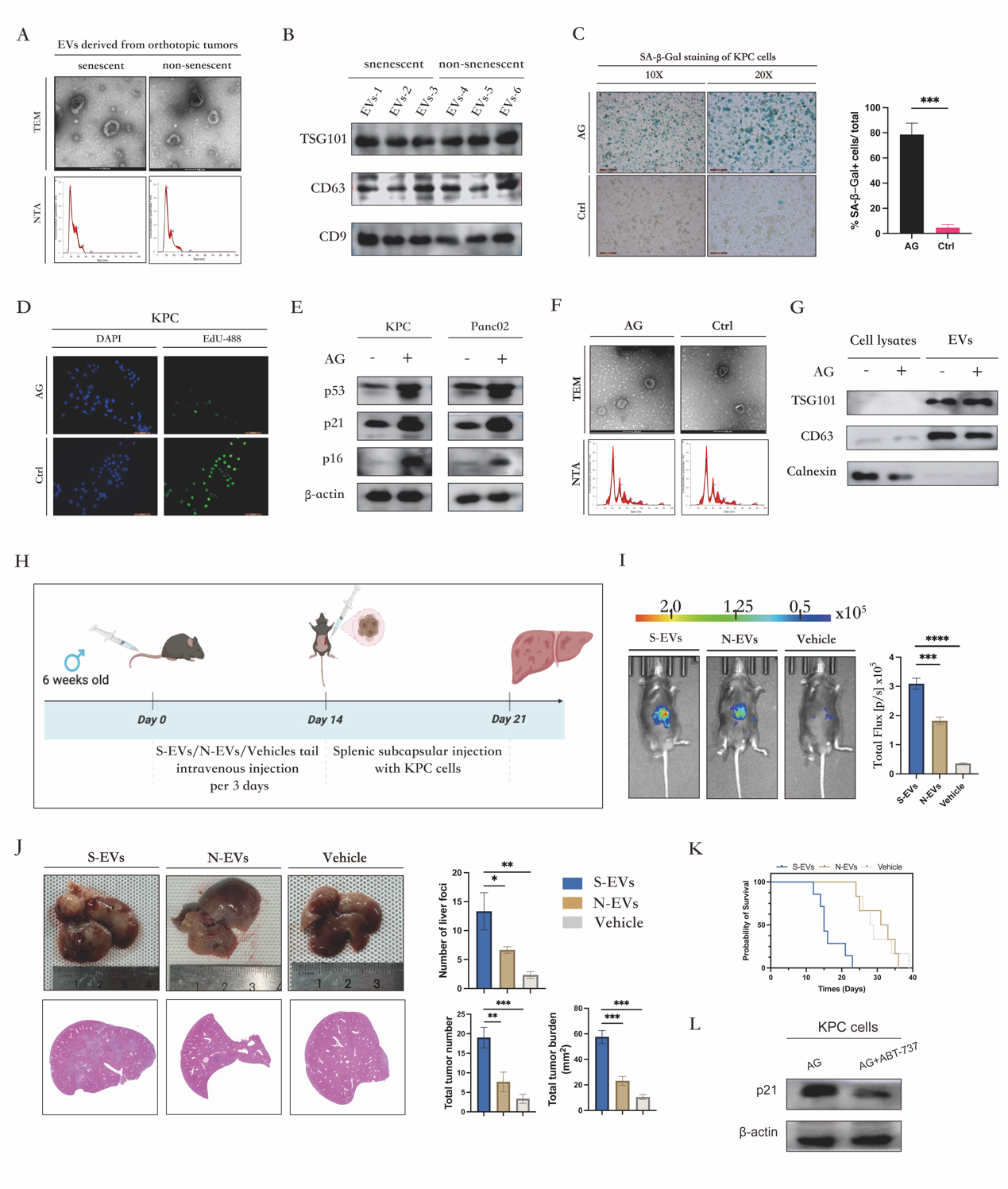
**

**Supplementary Figure.2 Characterization of chemotherapy-induced senescence and EV-mediated metastasis in vivo.**

(A–B) Characterization of EVs derived from orthotopic tumors of mice in the senescent and non-senescent groups. Representative transmission electron microscopy (TEM) images, nanoparticle tracking analysis (NTA), and western blot analysis of positive and negative EV markers are shown.

(C) Representative images of SA-β-gal staining and quantification of senescence in KPC cells treated with chemotherapy.

(D) Evaluation of KPC cell proliferation capacity using the EdU incorporation assay.

(E) Western blot analysis of p16, p21, and p53 expression in KPC and Panc02 cells treated with or without AG.

(F–G) Characterization of EVs derived from chemotherapy-treated (S-EVs) or vehicle-treated (N-EVs) KPC cells. TEM images, NTA profiles, and western blot analysis of EV markers are presented.

(H) Schematic illustration of the experimental workflow for the liver metastasis model following pre-treatment with S-EVs, N-EVs, or vehicle (n=6 per group).

(I) Representative in vivo bioluminescence imaging of liver metastasis in the S-EVs, N-EVs, and vehicle groups.

(J) Representative macroscopic images and H&E staining of liver tissues from the indicated groups.

(K) Kaplan–Meier survival curves showing overall survival (OS) of mice in the S-EVs, N-EVs, and vehicle groups.

(L) Western blot analysis of p21 expression in KPC cells (AG vs. AG + ABT-737).

**
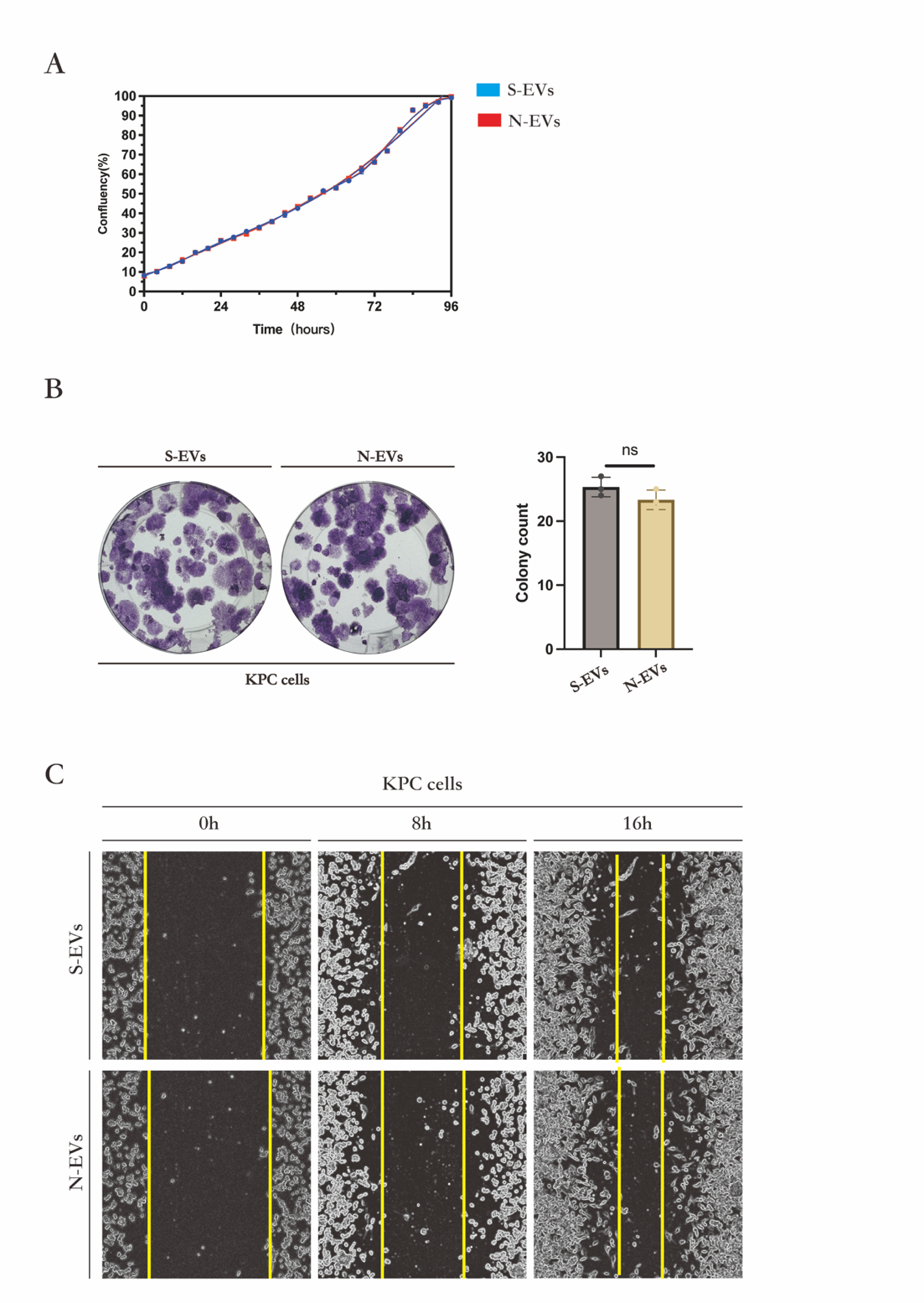
**

**Supplementary Figure.3 S-EVs do not affect the proliferative or migratory capacity of PDAC cells in vitro.**

(A) Proliferation curves of KPC cells treated with S-EVs or N-EVs, monitored by a live-cell imaging system.

(B) Representative crystal violet staining images (left) and quantification (right) of KPC cells following treatment with S-EVs or N-EVs.

(C) Representative images from the wound healing assay of KPC cells treated with S-EVs or N-EVs at 0, 8, and 16 hours.

**
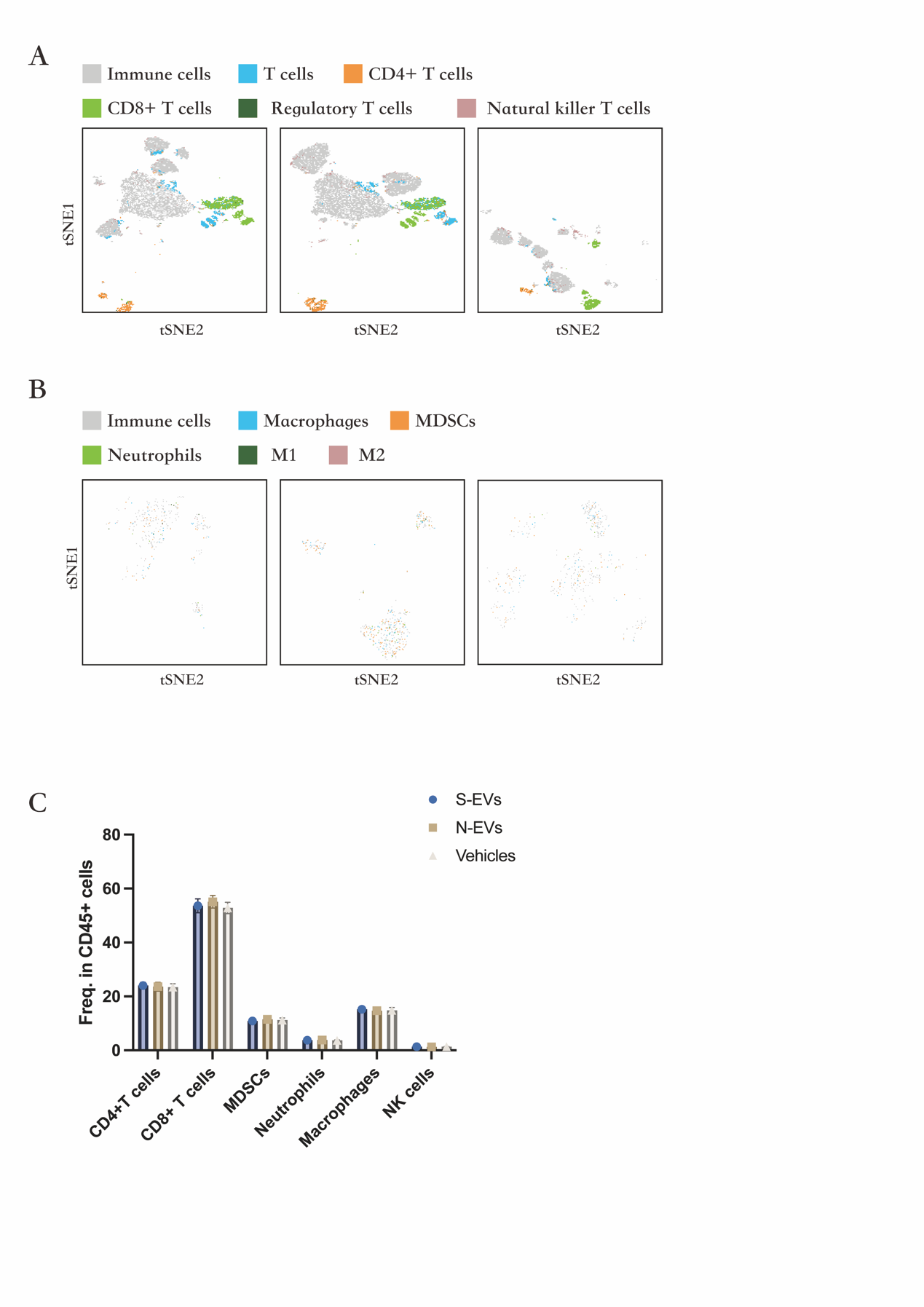
**

**Supplementary Figure.4 Immune landscape of liver metastases following EV treatment.**

(A–B) t-SNE analysis of flow cytometry data derived from liver metastases of mice treated with S-EVs, N-EVs, or vehicle.

(C) Flow cytometric quantification of the proportions of the indicated immune cell subsets (gated on CD45⁺ cells) in liver metastases from the S-EVs, N-EVs, and vehicle groups.

**Supplementary Figure.5 Flow cytometry analysis of orthotopic pancreatic tumor following S-EVs or N-EVs treatment.**

(A) Flow cytometric quantification of cytotoxic activation markers GZMB and IFN-γ on infiltrating CD8⁺ T cells from orthotopic pancreatic tumor treated with S-EVs or N-EVs.

**
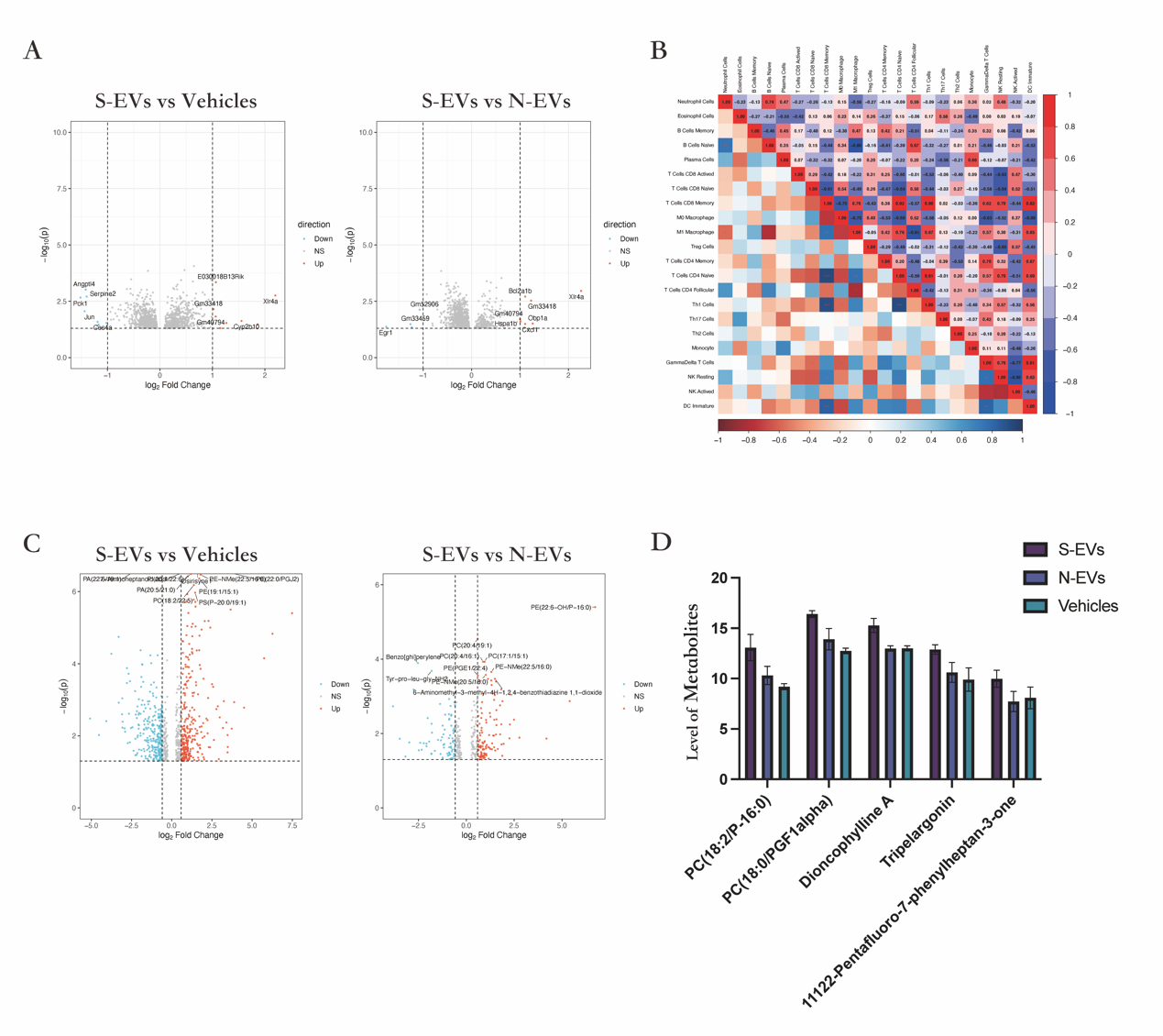
**

**Supplementary Figure.6 Transcriptomic and metabolomic profiling of liver tissues from mice treated with S-EVs, N-EVs, or vehicles.**

(A) Volcano plots of RNA-sequencing data illustrating differentially expressed genes (DEGs) in liver tissues. Comparisons of S-EVs vs. vehicle (left) and S-EVs vs. N-EVs (right) are shown.

(B) CIBERSORT analysis revealing the landscape of immune cell infiltration and their correlation matrix in liver tissues from the indicated groups.

(C) Volcano plots of metabolomic profiles comparing S-EVs vs. vehicle (left) and S-EVs vs. N-EVs (right).

(D) Quantification of the top 5 significantly altered metabolites.

**
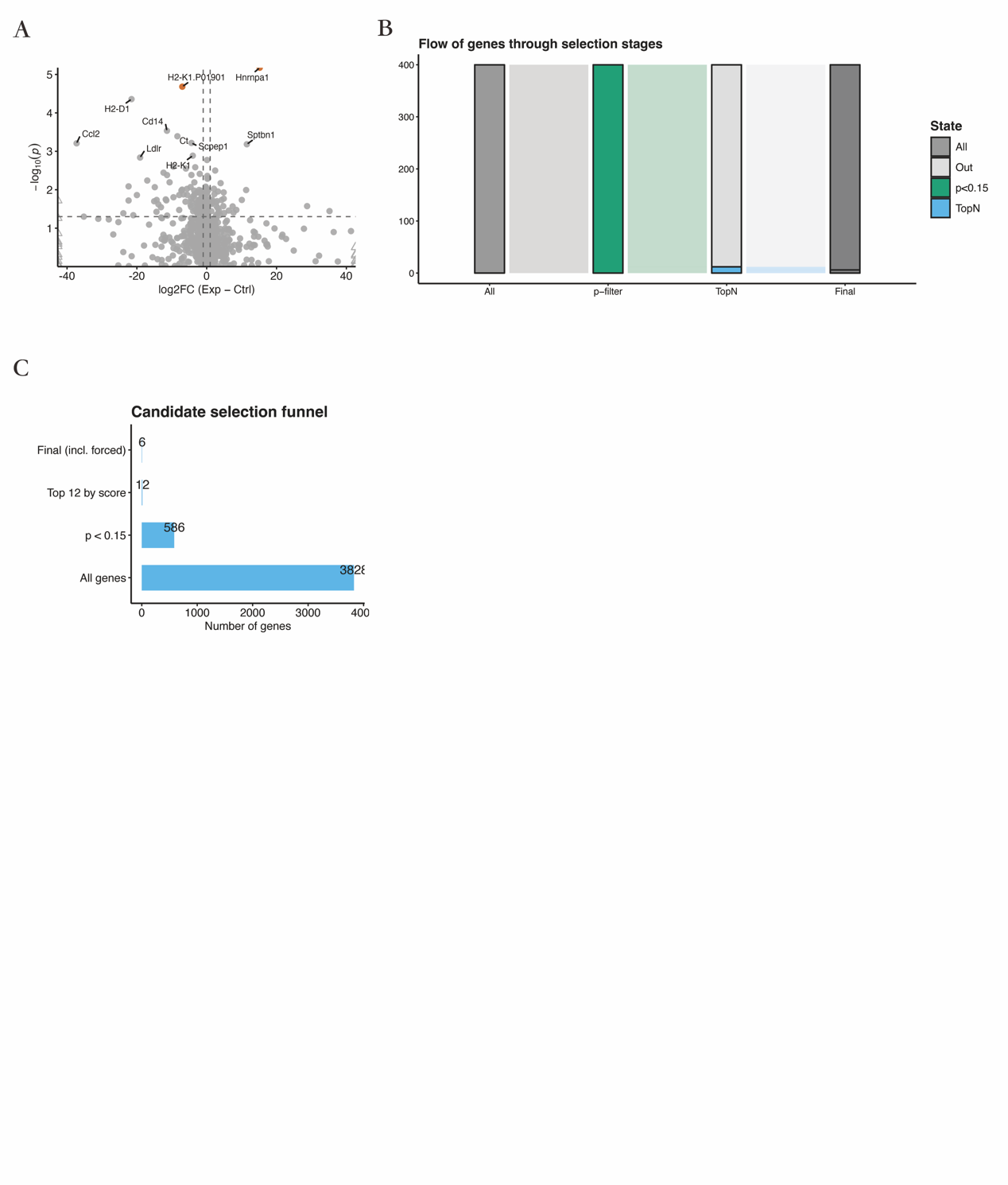
**

**Supplementary Figure.7 Identification of key senescence-associated proteins in S-EVs via proteomic analysis and functional screening.**

(A) Volcano plot of the proteomic data comparing the protein cargo of S-EVs versus N-EVs. Significantly enriched proteins in S-EVs are highlighted.

(B–C) Functional screening of candidate proteins using a "leave-one-out" strategy.

**Supplementary Figure.8 Expression of AChE** **in pancreatic tissues from mouse treated with S-EVs or N-EVs.**

(A) Western blot analysis of AChE expression in pancreatic tissues from mouse treated with S-EVs or N-EVs.

**
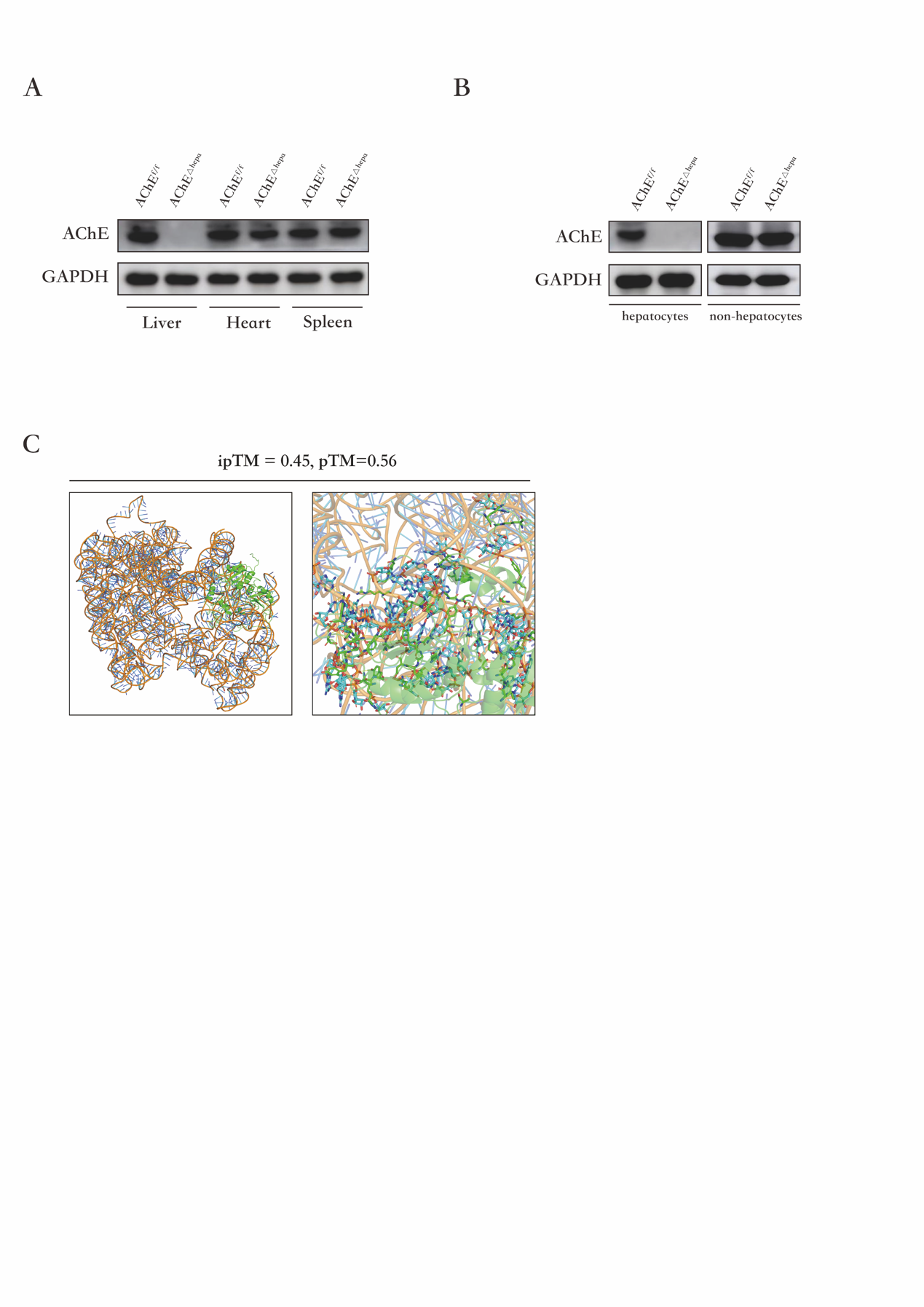
**

**Supplementary Figure.9 Validation of hepatocyte-specific AChE knockout and prediction of hnRNPA1-AChE interaction.**

(A) Western blot analysis of AChE expression in the liver, heart, and spleen tissues from AChE^f/f^ and AChE^△hepa^ mice.

(B) Western blot analysis of AChE expression in primary hepatocytes and non-hepatocyte fractions isolated from AChE^f/f^ and AChE^△hepa^ mice.

(C) Molecular docking model predicting the interaction between hnRNPA1 protein and AChE mRNA, generated using AlphaFold 3.


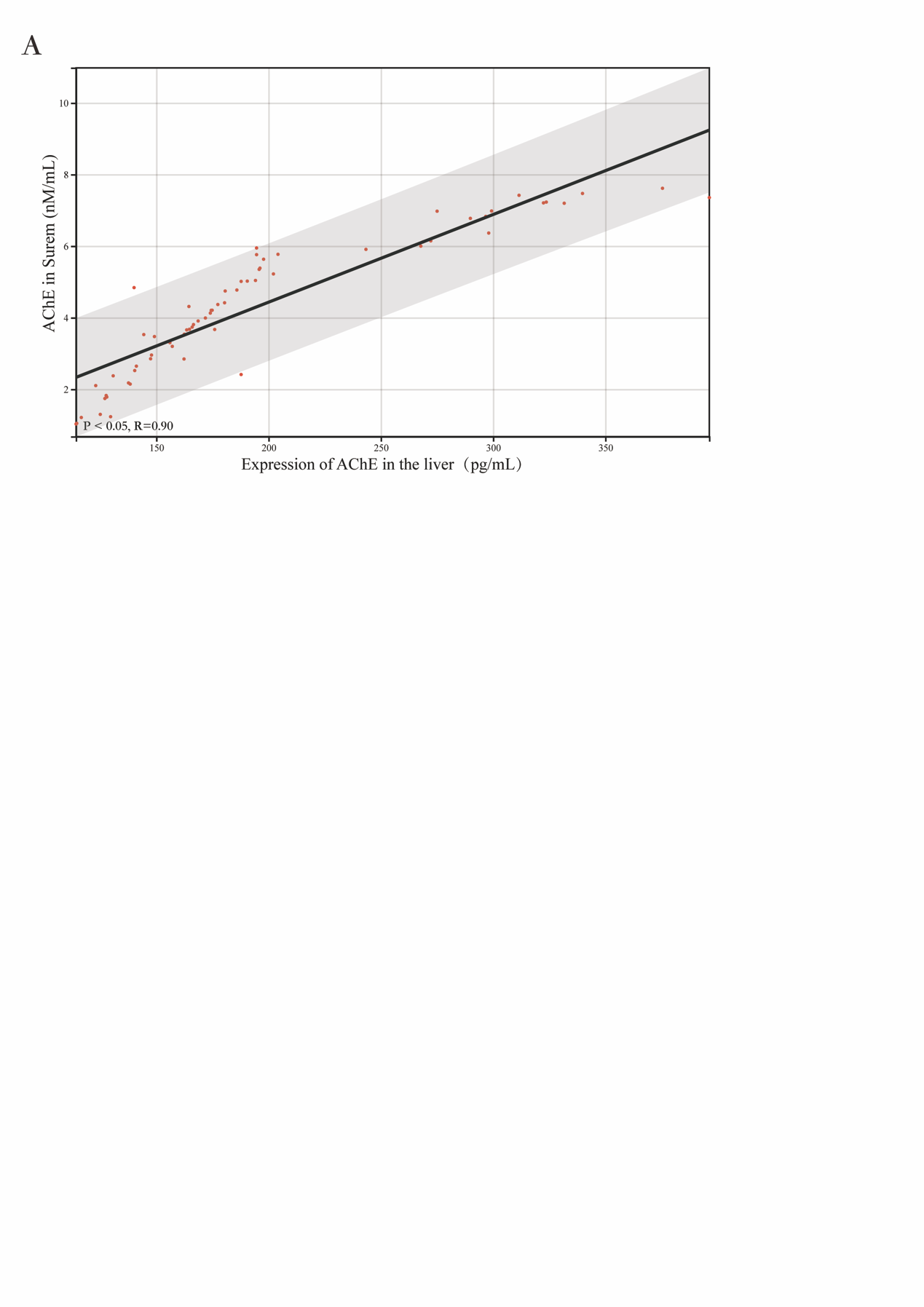


**Supplementary Figure.10 Positive correlation between hepatic and serum AChE levels in patients treated with neoadjuvant chemotherapy.**

(A) Pearson correlation analysis comparing AChE protein levels in resected liver metastatic tissues and matched serum samples from patients who underwent hepatectomy following neoadjuvant chemotherapy.
